# Screening Stability, Thermochemistry, and Chemical Kinetics of 3-Hydroxybutanoic Acid as a Bifunctional Biodiesel Additive

**DOI:** 10.1021/acs.jpca.4c01338

**Published:** 2024-05-10

**Authors:** Mohamed A. Abdel-Rahman, Abolfazl Shiroudi, Jacek Czub, Hao Zhao

**Affiliations:** † Chemistry Department, Faculty of Science, Suez University, Suez 43518, Egypt; ‡ Department of Physical Chemistry, Gdańsk University of Technology, Narutowicza 11/12, Gdańsk 80-233, Poland; § BioTechMed Center, Gdańsk University of Technology, Gdańsk 80-233, Poland; ∥ College of Engineering, Peking University, Beijing 100871, China

## Abstract

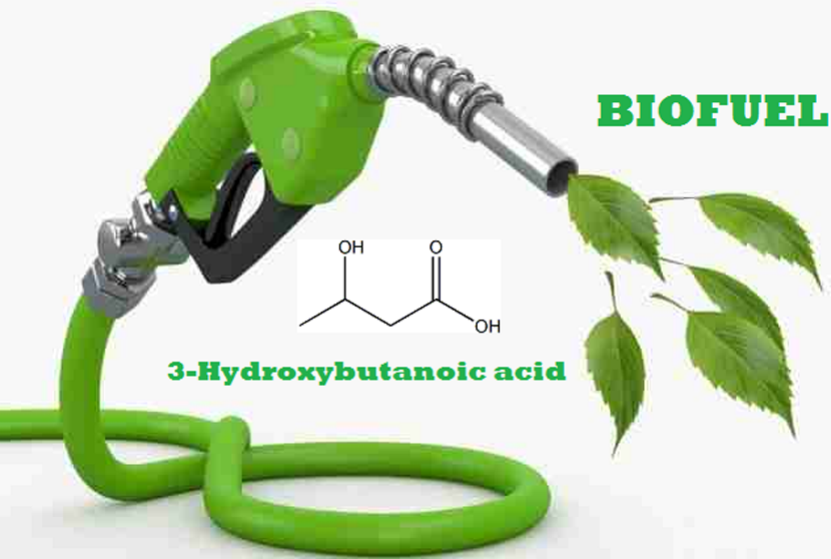

The thermo-kinetic aspects of 3-hydroxybutyric acid (3-HBA) pyrolysis in the gas phase were investigated using density functional theory (DFT), specifically the M06-2X theoretical level in conjunction with the cc-pVTZ basis set. The obtained data were compared with benchmark CBS-QB3 results. The degradation mechanism was divided into 16 pathways, comprising 6 complex fissions and 10 barrierless reactions. Energy profiles were calculated and supplemented with computations of rate coefficients and branching ratios over the temperature range of 600–1700 K at a pressure of 1 bar using transition state theory (TST) and Rice–Ramsperger–Kassel–Marcus (RRKM) methods. Thermodynamics results indicated the presence of six stable conformers within a 4 kcal mol^–1^ energy range. The estimated chemical kinetics results suggested that TST and RRKM approaches are comparable, providing confidence in our calculations. The branching ratio analysis reveals that the dehydration reaction pathway leading to the formation of H_2_O and CH_3_CH=CHCO_2_H dominates entirely at *T* ≤ 650 K. At these temperatures, there is a minor contribution from the simple homolytic bond fission reaction, yielding related radicals [CH_3_^•^CHOH + ^•^CH_2_CO_2_H]. However, at *T* ≥ 700 K, this reaction becomes the primary decomposition route. At *T* = 1700 K, there is a minor involvement of a reaction pathway resulting in the formation of CH_3_CH(OH)^•^CH_2_ + ^•^CHO(OH) with an approximate contribution of 16%, and a reaction leading to [^•^CH_3_ + ^•^CH_2_OHCH_2_CO_2_H] with around 9%.

## 1 Introduction

Biofuel represents a crucial form of renewable energy with the capacity to tackle fundamental worldwide challenges like environmental pollution and energy shortages. Despite biofuels leading to the release of greenhouse gases when consumed, they are considered carbon-neutral fuels, making them environmentally friendly.^1−3^ One specific biofuel variant, biodiesel, consists of alkyl ester fatty acids with long chains (12–20 carbon atoms) derived from biomass, including plants and animals.^4,5^

Bifunctional organic compounds are recognized as significant substitutes for energy sources due to their multifunctional groups, which can enhance various aspects of their ignition characteristics compared to unifunctional counterparts.^6,7^ One notable bifunctional organic compound is 3-hydroxybutyric acid (3-HBA), possessing both a hydroxyl group (–OH) and a carboxylic group (–COOH). This natural compound is present in human livers through the metabolism of fatty acids^8^ and is found in various organisms, such as the bacteria *Vitis rotundifolia* and *Cupriavidus necator*, among others. 3-HBA holds promise as a precursor for diverse biodegradable plastics, including polyester. In nature, bacteria like *Alcaligenes eutrophus* produce poly(3-hydroxybutyrate) from 3-hydroxybutyric acid.^9^ On a commercial scale, 3-HBA can be derived from poly(3-hydroxybutyrate) through acid hydrolysis.^10^

Despite challenges such as experimental shortages and high computational costs, there have been some experimental and computational studies focused on understanding the behavior of real biofuels and biodiesel molecules. These studies have provided valuable insights into the properties and performance of biodiesel.^6,7,11−33^ To comprehend the thermal degradation mechanism of real biodiesel, model biodiesel becomes essential. 3-HBA is regarded as a highly effective model for hydroxycarboxylic acid as a molecular biodiesel additive.

A computational study by Jin-bao et al.^32^ at the B3LYP/cc-pVTZ theoretical level investigated the decomposition mechanism through the elimination of CO and CO_2_ from 2,3,4-hydroxyl-butyraldehyde and 2,3,4-hydroxybutyric acid. Thermo-kinetic parameters were estimated for all pathways at various temperatures. The outcomes revealed six complex fission reactions (three for each compound), with the decarbonylation (CO elimination) from 2,3,4-hydroxybutyraldehyde being exothermic, while the decarboxylation (CO_2_ elimination) from 2,3,4-hydroxybutyric acid was endothermic. Notably, the direct activation energy for decarbonyl elimination was much lower than that occurring after dehydration, while for the decarboxyl reaction, the activation energy for decarboxyl elimination after dehydration was much lower than that occurring directly.

The theoretical mechanism of gas-phase pyrolysis of 4-bromobutyric acid to produce butyrolactone and hydrogen bromide was investigated by Tosta et al.^34^ The authors used both Møller–Plesset perturbation theory of second order (MP2) and density functional theory (DFT) at the PBE/6-31++G(d,p) level of theory to predict the reaction path. Their findings indicated a unimolecular reaction mechanism where the hydroxyl oxygen of the carboxylic group played a role in facilitating bromide removal through nucleophilic substitution. In a separate experimental study, Namysl et al.^35^ explored the oxidation of butanoic (butyric) and pentanoic (valeric) acids in a jet-stirred reactor under highly diluted conditions at temperatures ranging from 800 to 1100 K and a pressure of 800 Torr. The results revealed a broad spectrum of released products, starting from CO and CO_2_ molecules to C5 compounds, including 18 species for butanoic acid and 36 species for pentanoic acid.

To date, there has been no exploration, either computational or experimental, of the pyrolysis of 3-HBA as a bifunctional biodiesel under optimal combustion conditions. To address this gap, we utilize the M06-2X/cc-pVTZ theoretical level.^36,37^ Subsequently, we compare our calculated reaction energies and energy barriers with high-level composite CBS-QB3 results. The kinetics are assessed using transition state theory (TST)^38−41^ at the high-pressure limit, while the falloff behavior is analyzed statistically analysis employing the Rice–Ramsperger–Kassel–Marcus (RRKM) theory^42−44^ at lower pressures across a temperature range from 600 to 1700 K. Finally, to gain further insights into the pathways studied, we investigate into the results obtained from natural bond orbital (NBO) analysis.^45,46^

## 2 Computational Details

### 2.1 Potential Energy Surface (PES) Calculations

All quantum chemistry calculations were conducted with the Gaussian 09 suite of programs,^47^ and the molecular structures were visually analyzed with the ChemCraft package.^48^ The geometrical structures and vibrational frequencies of the parent molecule, 3-hydroxybutyric acid, transition states (TSs), and products were optimized using the DFT computational hybrid meta generalized gradient M06-2X functional,^37^ along with the correlation-consistent polarized valence triplet ζ (cc-pVTZ) basis set.^36^ To validate the obtained results at the M06-2X/cc-pVTZ theoretical level, a more accurate energy calculation was performed using the multilevel moderate computational cost CBS-QB3 composite method.^49−51^ The approach employed in this method includes low-level calculations on large basis sets, medium basis sets for second-order Møller–Plesset (MP2) calculations, and small basis sets for high-level correlation corrections [all coordinates are detailed in Table S1 in the Supporting Information]. The five-step CBS-QB3 series of calculations initiates with a geometry optimization at the B3LYP/6-311G(2d,d,p) level, followed by a frequency calculation to acquire thermal corrections, zero-point vibrational energy, and entropic information.^52−54^

The optimized structures were also confirmed to be real minima by frequency calculations. Frontier molecular orbital (FMO) properties and natural bond orbital (NBO) analysis are measured using the NBO technique. The molecular properties such as electronegativity (χ), chemical potential, ionization potential (IP), chemical hardness (η), softness (ζ), and global electrophilicity index (ψ) were calculated using highest occupied molecular orbital–least unoccupied molecular orbital (HOMO–LUMO) analysis at the same theoretical level using the NBO 5.0 program.^55^

To check the nature of different complex fission TSs, the minimum energy path^56^ was determined using intrinsic reaction coordinate (IRC) calculations^57,58^ at the M06-2X/cc-pVTZ level of theory. The IRC calculations were performed in both directions (forward and reverse) with 20 points, employing a step size of 0.1 amu^1/2^ Bohr.

### 2.2 Calculation of Absolute Rate Constants

**Scheme 1:**
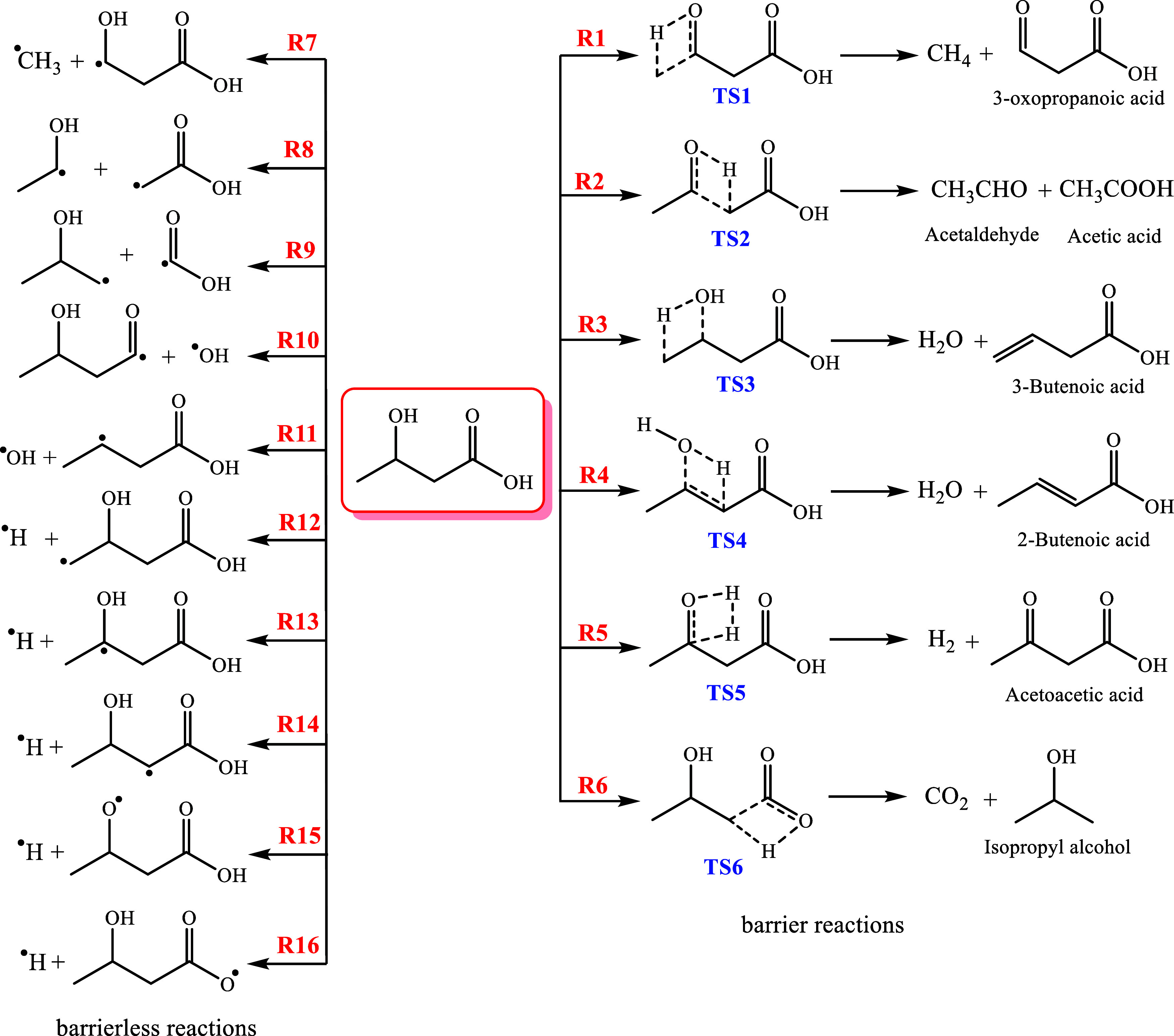
Possible Decomposition Chemical Channels of 3-HBA

According to Scheme 1, the pyrolysis mechanism of 3-hydroxybutyric acid involves two kinds of chemical pathways: (a) barrier reactions, which include hydrogen atom transfer [**R1**–**R6**], and (b) barrierless reactions of simple bond cleavage through reaction pathways **R7**–**R16**.

#### 2.2.1 Chemical Kinetic of Barrier Reactions

In the statistical adiabatic channel model (SACM), this frequent observation was attributed to the appearance of adiabatic channel energy barriers, which are a consequence of the interplay of the radial and angular parts of the interaction potential and which, by analogy with centrifugal barriers, contract the available phase space.^59−63^ In the statistical adiabatic channel model, adiabatic channel potential curves *V*_*i*_(*r*) are calculated. Their maxima define the channel threshold energies *E*_o*i*_ which, for thermal conditions, lead to the “activated complex” partition function^62−64^
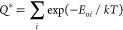
1using *E*_o*i*_ or Q*, the usual formalism of statistical rate theory (such as TST) is used. For very low temperatures, only the lowest channel states contribute. The potential curves of these channels can be obtained analytically from perturbation theory such that analytical expressions for channel threshold energies, activated complex partition functions, and capture rate coefficients can be obtained as well.^62,63^ To get accurate chemical kinetics, the KiSThelP program^65^ was employed to compute the rate coefficients of unimolecular barrier reactions [**R1**–**R6**], denoted as *k*_uni_ (in s^–1^) utilizing transition state theory as follows:
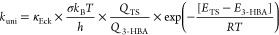
2where σ is the reaction pathway degeneracy, κ_Eck_(*T*) denotes one-dimensional Eckart correction tunneling,^66^ and *k*_B_ and *h* are the Boltzmann and Planck constants, respectively. In the above equations, *Q*_3-HBA_, and *Q*_TS_ represent the total molecular partition functions for 3-HBA and the transition state, respectively. The energy corresponding to these functions [*E*_3-HBA_ and *E*_TS_], including zero-point vibrational contributions, is calculated using Eckert’s tunneling correction at different temperatures as follows:
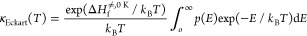
3where Δ*H*_f_^≠,0 K^ represents the zero-point corrected energy barriers in the forward direction and *p*(*E*) denotes the probability of transmission through the one-dimensional barrier at energy *E*. Atmospheric pressures are considered sufficient for reliably calculating the kinetics rate constant using TST. Additionally, the falloff behavior of canonical kinetic rate constants, denoted as *k*(*T*), transitioning from the TST limit (*P* → ∞) toward the low-pressure limit (*P* → 0), is computed using the RRKM theory. The microcanonical kinetics, represented by *k*(*E*), are assessed according to unimolecular RRKM theory^44^
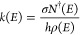
4where ρ(*E*) represents the vibrational density of states of the reactants and *N*^†^(*E*) denotes the total number of states at the transition state. The canonical rate constant *k*(*T*) is defined by^67^
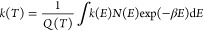
5where *Q*(*T*) represents the internal partition functions of the reactants and β denotes the Boltzmann constant (β = 1/*k*_B_*T*). All supplied kinetic data using TST and RRKM theories were obtained using the KiSThelP program. TST provides an upper limit estimate for rate constants in the high-pressure limit,^67^ and RRKM evaluates pressure effects on a microcanonical basis. Collisional stabilization rate constants were computed using Lennard-Jones (L-J) collision rate theory, and the effective collision frequency is given by the following equation:^68^
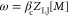
6where β_c_ represents the collisional efficiency, *Z*_L-J_ is the Lennard-Jones (L-J) collision frequency, and [M] denotes the concentration of the buffer gas.^69^ The retained value for β_c_ is 0.2.^65^ The collision frequencies (*Z*_LJ_) were calculated using the collisional L-J parameters (σ, ε/*k*_B_) obtained from the Joback method, which depends on the energy depth (ε) of the L-J potential and σ, representing a dimensional scale of the molecular radius.^70^ For helium as a diluent gas, the retained Lennard-Jones potential parameters are σ = 2.64 Å and ε/*k*_B_ = 10.9 K, while for 3-HBA, σ = 6.26 Å and ε/*k*_B_ = 550.91 K.^71^

#### 2.2.2 Chemical Kinetic of Barrierless Reactions

To calculate the rate coefficient of barrierless reactions, the accurate classical method was used. The classical method was tested recently for many comparable systems and gave accurate results.^6,29−31,72,73^

## 3 Results and Discussion

### 3.1 3-Hydroxybutyric Acid Conformers

**Figure 1:**
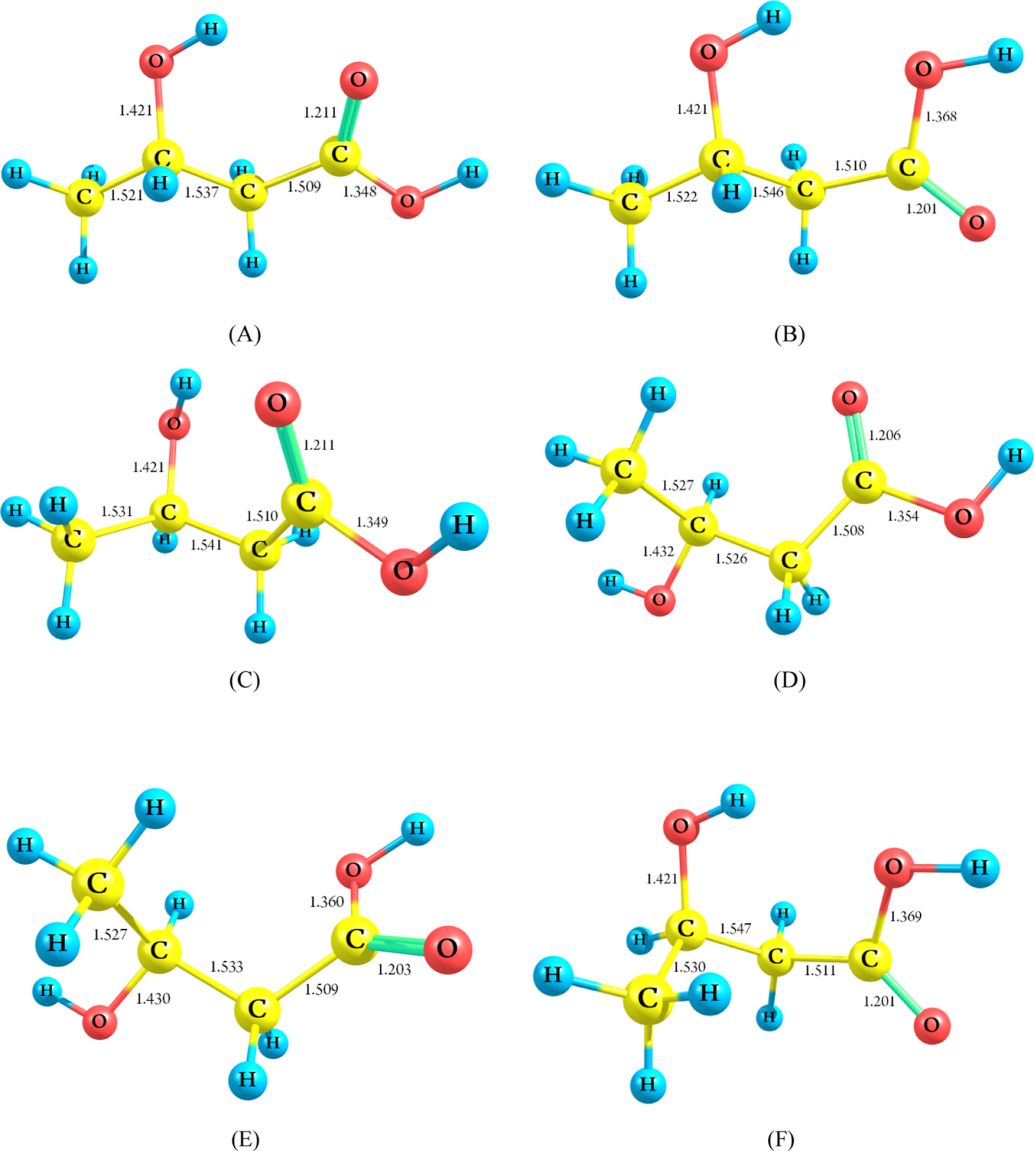
Optimized structures for the different 3-HBA conformers at the CBS-QB3 method.

**Figure 2:**
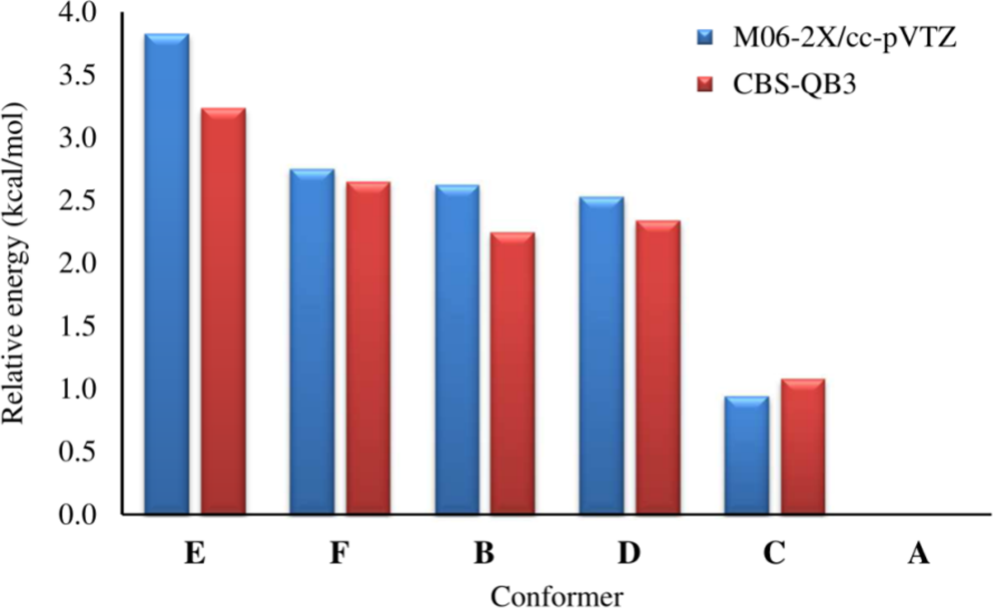
Relative energies of 3-HBA conformers at the M06-2X/cc-pVTZ and CBS-QB3 methods.

Due to the rotation of the terminal methyl group (–CH_3_), the internal hydroxyl group, and the carboxyl group, the **3-HBA** molecule exhibits six stable conformers: **A**, **B**, **C, D, E**, and **F**. The optimized structures of the **3-HBA** conformers and their relative energies are depicted in Figures 1 and 2, respectively.

The sum of electronic and zero-point energies as well as the relative energy (Δ*E*), was investigated in the gas phase, and the results are summarized in Table S2 in the Supporting Information. Upon examination of these conformers, it is evident from the relative energies that conformer **A** is the most stable, while conformer **E** is the least stable, with energies of 3.22 (3.24) kcal mol^–1^, respectively, at the M06-2X and CBS-QB3 (in parentheses) levels relative to conformer **A** (see Figure 2 and Table S2 in the Supporting Information). Conformers **B**, **C**, **D**, **E**, and **F** exhibit energies of 2.62 (2.25), 0.95 (1.08), 2.53 (2.34), 3.83 (3.24), and 2.75 (2.65) kcal mol^–1^, respectively, relative to conformer **A**.

### 3.2 Frontier Molecular Orbital Analysis

**Figure 3:**
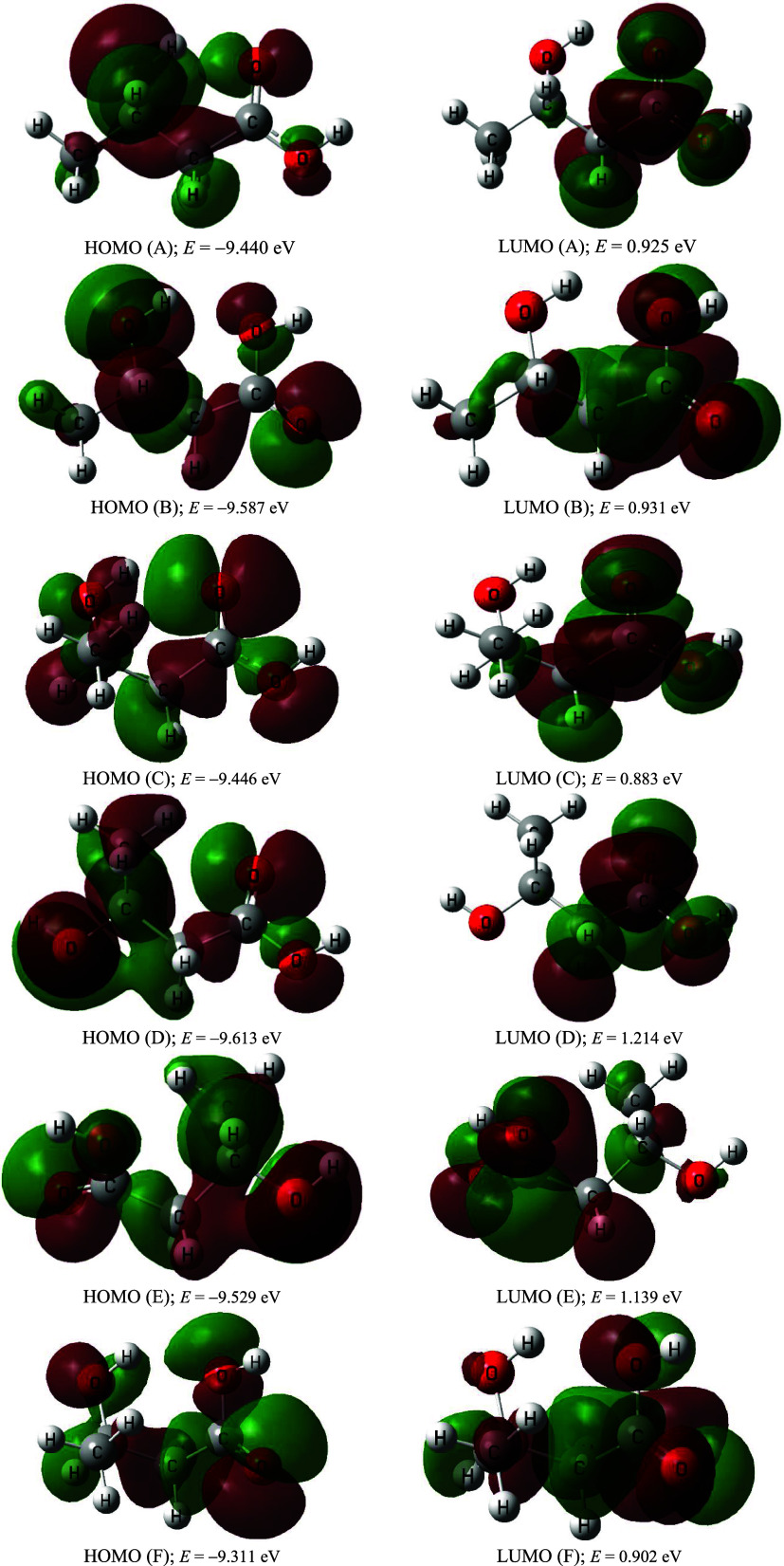
Representative molecular structures of the HOMO and LUMO orbitals of 3-HBA conformers calculated at the M06-2X/cc-pVTZ level of theory.

**Figure 4:**
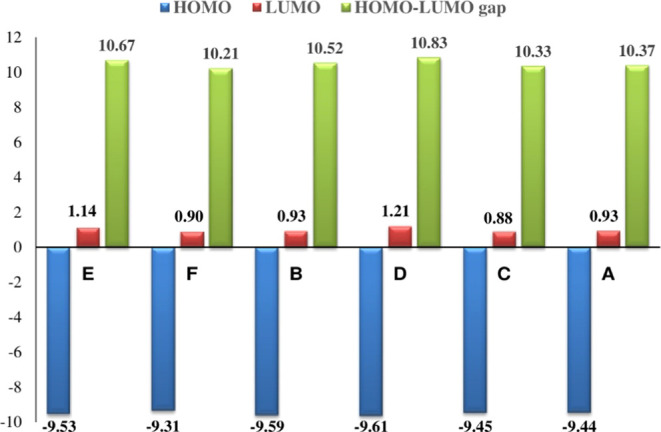
Energy levels of HOMO and LUMO, as well as the HOMO–LUMO energy gaps (in eV) of 3-HBA conformers at the M06-2X/cc-pVTZ level of theory.

Frontier molecular orbital (FMO) energies play a crucial role in determining the stability, reactivity, optical, and electrical properties of organic molecules. The HOMO–LUMO energy gap explains the concluding charge transfer interaction within the molecule and is useful in determining molecular electrical transport properties.^74,75^ The estimated LUMO and HOMO of the studied conformers give extensive explanations of their molecular electronic properties. The HOMOs and LUMOs plots at the M06-2X/cc-pVTZ level of theory are presented in Figure 3, while the corresponding HOMO–LUMO energy gaps at the studied methods are shown in Figure 4. The HOMO–LUMOs results indicate gaps of 10.37, 10.52, 10.33, 10.83, 10.67, and 10.21 eV for **A**, **B**, **C**, **D**, **E**, and **F** conformers, respectively. Based on the relative energy results, these conformers can be arranged as follows: **E >F> B > D > C > A**, while the stability order concerning HUMO–LUMOs energy gap is **F** < **C < A < B** < **E** < **D**.

This study calculates HOMO and LUMO energies using the same theoretical level, focusing on chemical hardness and polarizability. Analysis of the table reveals that conformer **D** (Δ*E* = 10.83 eV) is characterized as hard and more stable, indicating lower chemical reactivity. Conversely, conformer **F** (Δ*E* = 10.21 eV) is identified as soft and the least stable in the gas phase, signifying higher chemical reactivity.

### 3.3 Validity of the Studied DFT Method

**Figure 5:**
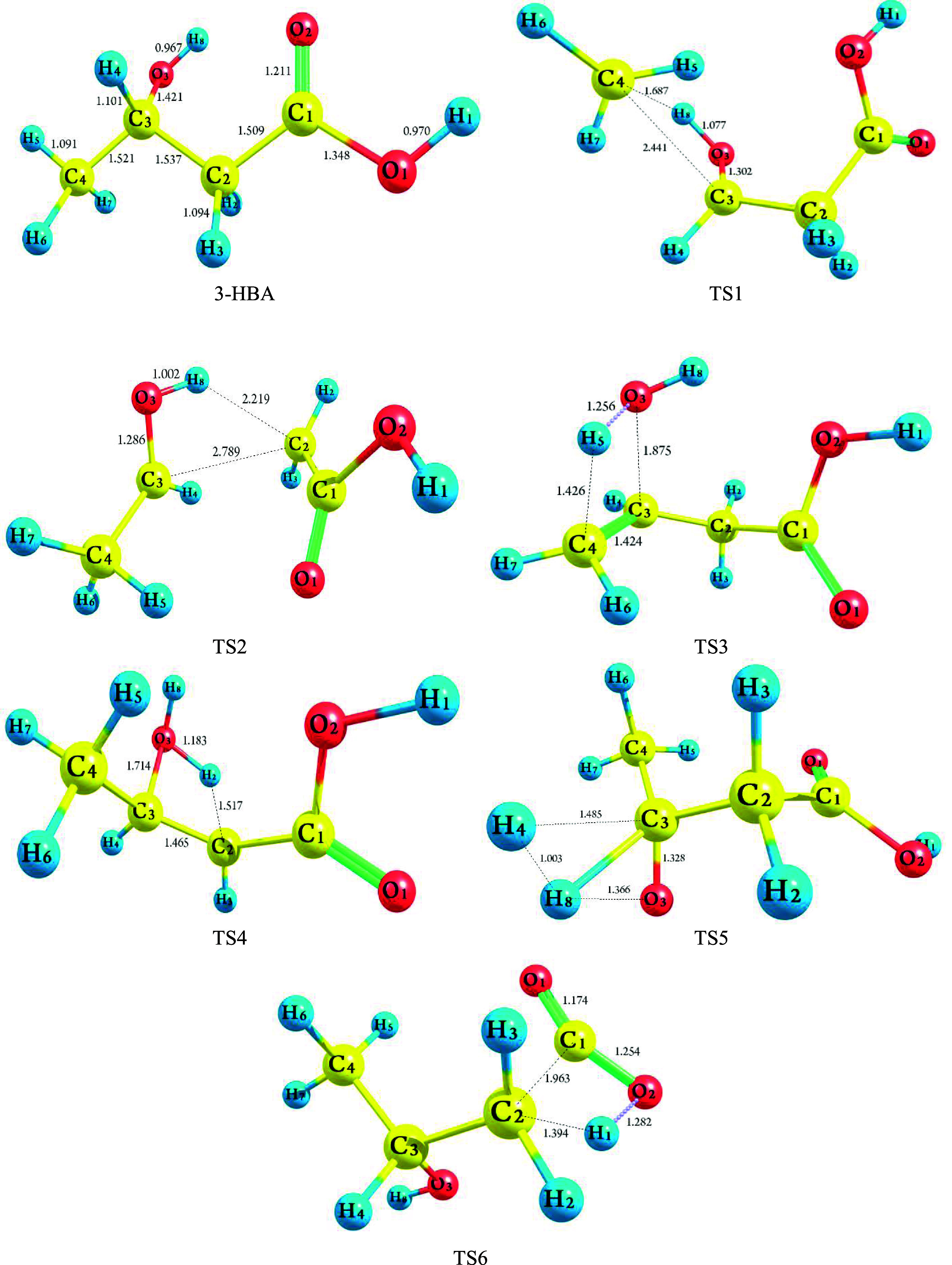
Optimized structure of the transition states at the CBS-QB3 method for unimolecular complex bond fission of 3-HBA [bond lengths are given in angstrom (Å)].

**Figure 6:**
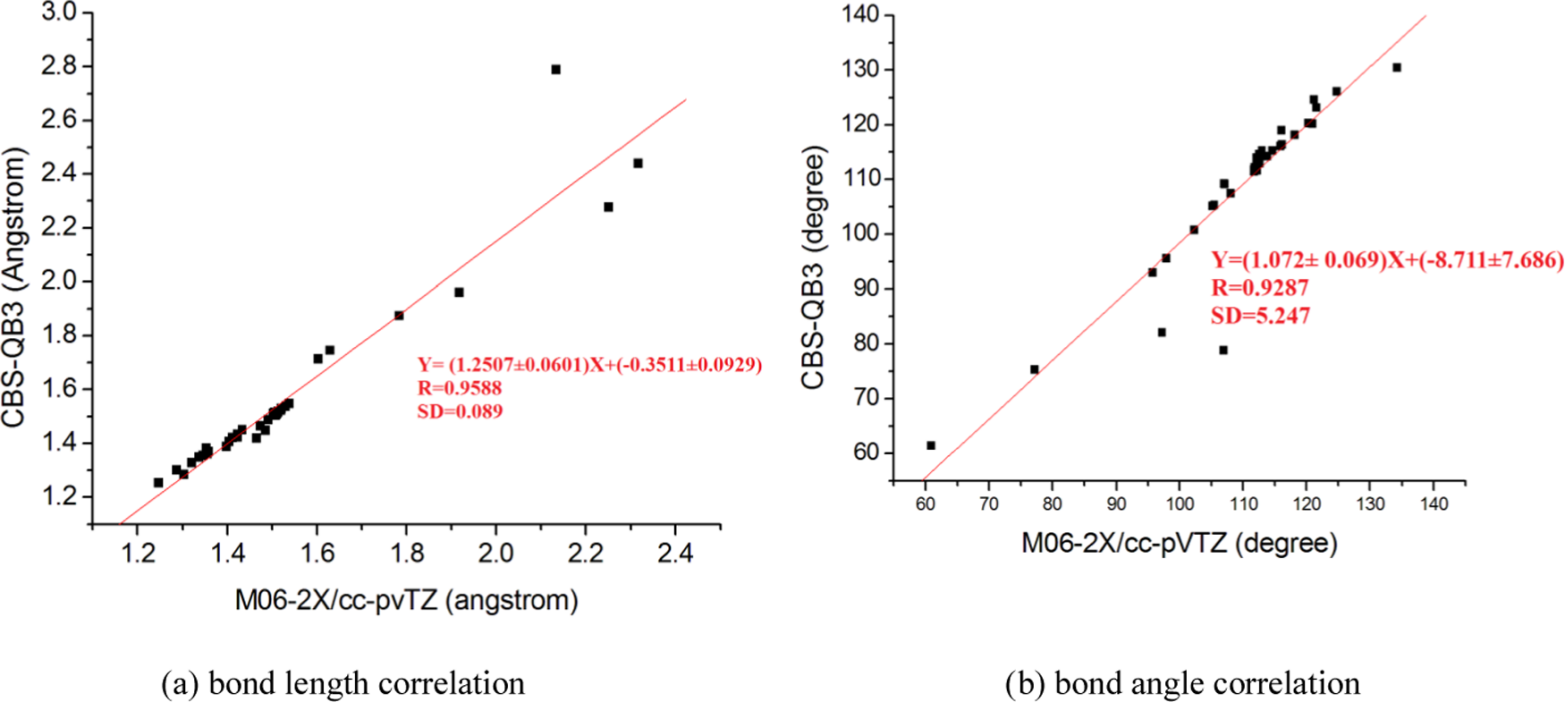
Correlation between CBS-QB3 and M06-2X/cc-pVTZ results: (a) bond lengths and (b) bond angles.

Figure 5 illustrates the optimized geometries of 3-HBA and its different transition states at the CBS-QB3 method. Meanwhile, Table S3 in the Supporting Information compiles the geometrical parameters of main bond lengths and angles obtained through the CBS-QB3 method. The correlation between the M06-2X/cc-pVTZ and CBS-QB3 results is depicted in Figure 6.

To assess the validity of the employed computational methods, the mean absolute error (MAE), mean signed error (MSE), and root-mean-square error (RMSE) have been computed. The expressions for MSE, MAE, and RMSE are as follows:
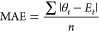
7
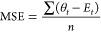
8
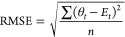
9where *n* represents the total number of observations, and θ_*t*_ and *E*_*t*_ denote the M06-2X/cc-pVTZ and CBS-QB3 results, respectively. Utilizing the previously mentioned equations for MSE, MAE, and RMSE calculations, the findings indicate the following discrepancies in bond lengths: 0.009, 0.021, and 0.026 Å for the C_1_–C_2_ bond; 0.083, 0.087, and 0.231 Å for the C_2_–C_3_ bond; 0.016, 0.025, and 0.046 Å for the C_3_–C_4_ bond; 0.023, 0.025, and 0.043 Å for the C_1_–O_2_ bond; and 0.029, 0.033, and 0.052 Å for the C_2_–O_3_ bond. Regarding bond angles, the variations are as follows: −1.82, 5.22, and 10.13° for the C_1_–C_2_–C_3_ angle; −4.30, 4.30, and 9.40° for the C_2_–C_3_–C_4_ angle; −2.13, 2.27, and 5.40° for O_3_–C_3_–C_2_ angle; and −0.70, 0.80, and 1.21° for O_3_–C_3_–C_4_ angle. These results demonstrate a substantial agreement between the CBS-QB3 and M06-2X/cc-pVTZ methods.

### 3.4 Thermochemistry of Complex Bond Fission Reactions

**Figure 7:**
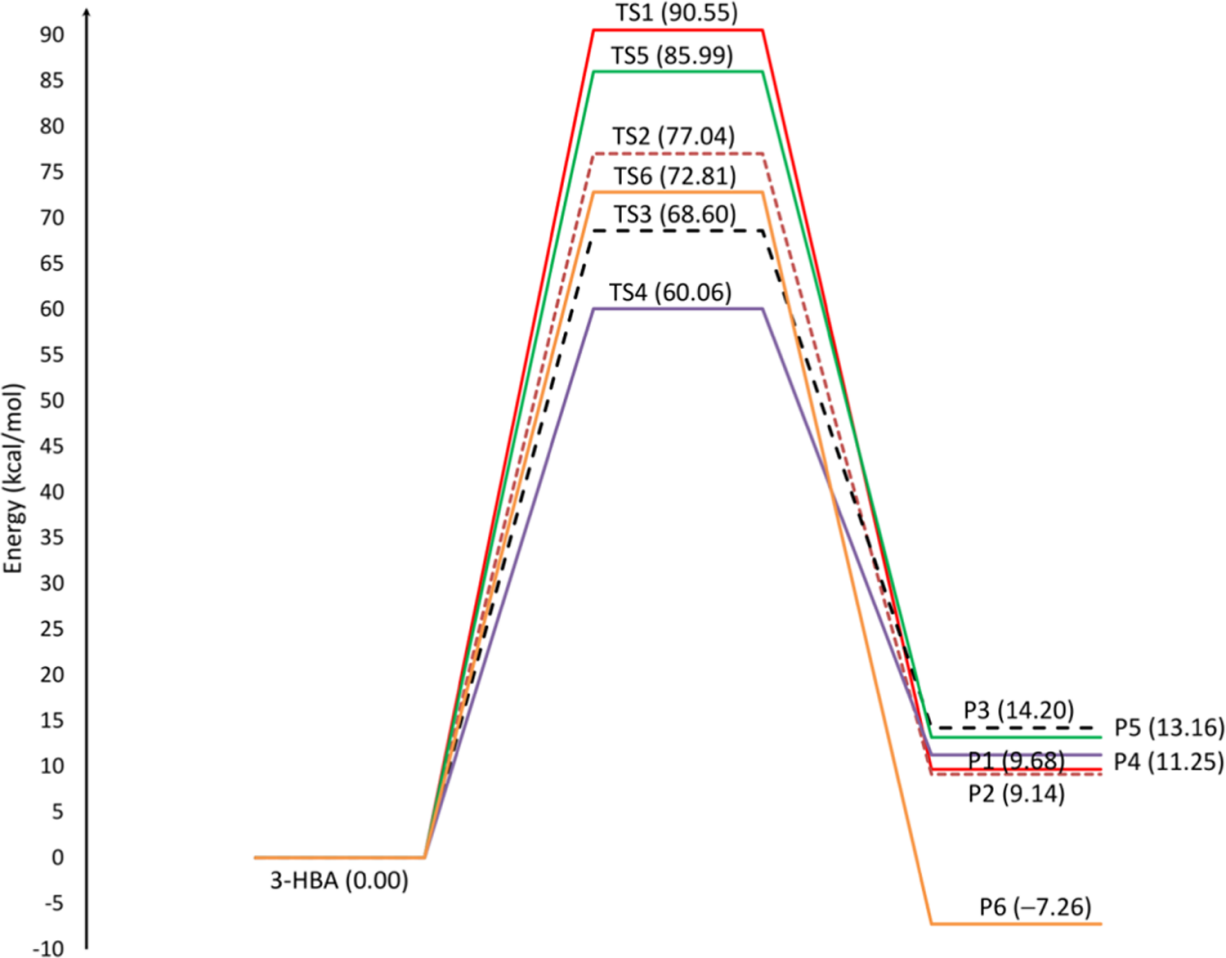
Potential energy profile for barrier reactions involved in the unimolecular degradation mechanism of 3-HBA (Δ*E*_298 K_, Δ*E*_298 K_^†^, in kcal mol^–1^) at the CBS-QB3 method (*T* = 298 K, *P* = 1 bar).

Complex fission (barrier) reactions refer to reactions that occur through hydrogen transfer or molecular elimination. According to Scheme 1, the 3-hydroxybutyric acid molecule can undergo pyrolysis through six complex fission reactions (**R1**–**R6**). The optimized structures of 3-HBA and the resulting transition states (**TS1**–**TS6**) are illustrated in Figure 5 and Table S3 in the Supporting Information, as well as with Cartesian structures in Table S1 in the Supporting Information. According to this figure, reactions **R1** and **R2** exhibit energy barrier reactions involving 1,3-H-transfer reactions through transition states **TS1** and **TS2**, resulting in the production of “methane and 3-oxo-propionic acid” and “acetaldehyde and acetic acid”, respectively. Dehydration (water elimination) of the parent molecule leads to the formation of 3-butenoic acid and 2-butenoic acid in reactions **R3** and **R4**, respectively. Additionally, acetoacetic acid can be obtained in reaction **R5** through the elimination of a hydrogen molecule via transition state TS5. Molecular elimination of CO_2_ occurs in reaction **R6** yielding isopropyl alcohol. Tables 1 and 2 provide a summary of the barrier and reaction energetic and thermodynamic parameters during the pyrolysis of 3-hydroxybutyric acid using the M06-2X and CBS-QB3 methods, and the corresponding potential energy diagram is presented in Figure 7. The variations in bond lengths and bond angles along the reaction coordinate for the generation of different products via reaction pathways **R1**–**R6** are illustrated in Figure S1 in the SI, utilizing both the M06-2X/cc-pVTZ and CBS-QB3 methods.

**Table 1:** Activation Parameters (Energy, Enthalpy, and Gibbs Free Energy) (in kcal mol^–1^) for Barrier Reactions Encounter 3-HBA Pyrolysis along with Pathways **1**–**6** at the Studied Methods (*P* = 1 atm)

method	M06-2X/cc-pVTZ			CBS-QB3		
species	Δ*E*_0 K_^†^	Δ*H*_298 K_^†^	Δ*G*_298 K_^†^	Δ*E*_0 K_^†^	Δ*H*_298 K_^†^	Δ*G*_298 K_^†^
3-HBA	0.00	0.00	0.00	0.00	0.00	0.00
3-HBA → TS1	95.27	95.29	95.21	90.55	90.74	90.25
imaginary frequency TS1 (cm^–1^)	1329.1*i*			968.5*i*		
3-HBA → TS2	80.54	80.89	79.66	77.04	77.39	76.30
imaginary frequency TS2 (cm^–1^)	2224.7*i*			1833.2*i*		
3-HBA → TS3	67.45	67.45	67.50	68.60	68.64	68.57
imaginary frequency TS3 (cm^–1^)	1919.8*i*			2025.6*i*		
3-HBA → TS4	58.24	58.10	58.70	60.06	60.16	59.78
imaginary frequency TS4 (cm^–1^)	1226.3*i*			1684.2*i*		
3-HBA → TS5	86.27	86.28	85.91	85.99	85.99	85.71
imaginary frequency TS5 (cm^–1^)	2246.9*i*			2218.3*i*		
3-HBA → TS6	71.58	71.87	71.11	72.81	73.11	72.40
imaginary frequency TS6 (cm^–1^)	1848.2*i*			2032.1*i*		

**Table 2:** Reaction Parameters (Energy, Enthalpy, and Gibbs Free Energy) (in kcal mol^–1^) of the 3-HBA Pyrolysis along with Pathways **1**–**6** at the Studied Methods (*P* = 1 atm)

method	M06-2X/cc-pVTZ			CBS-QB3		
species	Δ*E*_0 K_	Δ*H*°_298 K_	Δ*G*°_298 K_	Δ*E*_0 K_	Δ*H*°_298 K_	Δ*G*°_298 K_
3-HBA	0.00	0.00	0.00	0.00	0.00	0.00
3-HBA → pre-RC1	10.41	12.26	7.07			
3-HBA → CH_4_ + CHOCH_2_CO_2_H	11.50	12.93	0.25	9.68	11.09	–1.52
3-HBA → pre-RC2	5.55	7.22	1.54			
3-HBA → CH_3_CHO + CH_3_CO_2_H	10.21	10.82	–1.64	9.14	10.18	–3.26
3-HBA → pre-RC3	12.09	13.50	9.89			
3-HBA → H_2_O + CH_2_=CHCH_2_CO_2_H	17.22	18.83	6.79	14.20	15.81	3.82
3-HBA → pre-RC4	11.78	13.54	8.69			
3-HBA → H_2_O + CH_3_CH=CHCO_2_H	14.32	15.96	4.10	11.25	12.90	0.96
3-HBA → pre-RC5	16.86	18.32	14.08			
3-HBA → H_2_ + CH_3_CHOCH_2_CO_2_H	15.30	17.39	7.10	13.16	15.21	4.80
3-HBA → pre-RC6	–6.59	–5.43	–9.14			
3-HBA → CO_2_ + CH_3_CHOHCH_3_	–5.35	–4.57	–15.73	–7.26	–6.46	–17.55

Inspection of the B3LYP/6-311G(2d,d,p) geometries obtained for the transition state **TS1** along reaction pathway **1** (**R1**) reveals that the hydrogen atom from the hydroxyl group migrates to the terminal C_4_ of the CH_3_ (methyl) group, resulting in the production of methane and 3-oxopropionic acid. The reaction requires barrier heights of 90.55 kcal mol^–1^ and reaction energy of 9.68 kcal mol^–1^, as determined by the CBS-QB3 method. As depicted in Figure 5, investigation of the **TS1** structure shows that the elongation of the O_3_–H_8_ and C_3_–C_4_ bonds is evident, with increases of 0.110 Å (11.38%) and 0.920 Å (60.49%), respectively, compared to the equilibrium structure computed for 3-HBA. In contrast, the forming C_4_–H_8_ bond has a larger length than in the isolated methane molecule, and the C_3_=O_3_ bond length is shortened by 0.119 Å (8.37%).

Reaction **2** leads to the cleavage of the C_2_–C_3_ and O_3_–H_8_ bonds through the four-membered ring transition state **TS2** to produce acetaldehyde and acetic acid located at 9.14 kcal mol^–1^ above the 3-HBA at the CBS-QB3 method. TS2 results from a simple elongation of the breaking O_3_–H_8_ and C_2_–C_3_ bond lengths and the simultaneous shrinkage of the C_3_–O_3_ distance as a result of the forming of the C_2_–H_8_ simple bond. The C_2_–C_3_ and O_3_–H_8_ bonds are elongated by 2.789 and 1.002 Å, respectively, and the forming C_2_–H_8_ bond is shrunk by 2.219 Å. As depicted in Figure 5, the bond formation of the C_2_–H_8_ and C_3_=O_3_ is reduced by 1.127 Å (50.79%) and 0.082 Å (6.81%), respectively, while the bond breaking of the O_3_–H_8_ and C_2_–C_3_ undergoes stretching by 0.035 Å (3.62%) and 1.252 Å (81.46%), respectively.

The transition state **TS3** structure obtained along pathway **3** demonstrates that the oxygen atom (O_3_) of the hydroxyl group attracts the H_5_ atom of the terminal C_4_ atom (CH_3_ group). Conversely, in the case of the **TS4** structure, the O_3_ atom captures the hydrogen atom of the internal C_2_ atom of the CH_2_ group. Among all of the barrier reactions considered in this work, the elimination of a water molecule during chemical reaction **R4** is the most favorable kinetic route. The four-membered ring **TS4** structure, with an activation energy of 60.06 kcal mol^–1^, is energetically more favorable than the **TS3** structure by 8.54 kcal mol^–1^ and is less endothermic by 2.95 kcal mol^–1^, according to the CBS-QB3 method. Inspection of Figure 5 shows that during the progress of reaction **R3**, the forming O_3_–H_5_ bond has a larger length than in the isolated H_2_O molecule in the TS3 structure (0.294 Å), and the formation of the C_3_=C_4_ bond is shortened by 0.097 Å (6.38%). On the contrary, the breaking C_4_–H_5_ and C_3_–O_3_ bonds are elongated by 0.335 Å (30.71%) and 0.454 Å (31.95%), respectively. Furthermore, in the **TS4** structure, the bond lengths of O_3_–H_2_ and C_2_=C_3_ are shortened by 1.183 Å and 1.465 Å, respectively, while the bond breaking of C_2_–H_2_ and C_3_–O_3_ is elongated by 0.423 Å (38.67%) and 0.293 Å (20.62%), respectively.

In pathway **5**, acetoacetic acid can be obtained through the four-membered ring transition state TS5 when the hydrogen atom of the hydroxyl group joins with the hydrogen atom of the tertiary C_3_ atom. The reaction is achieved with a barrier height of 85.99 kcal mol^–1^ and a reaction energy of 13.16 kcal mol^–1^, according to the CBS-QB3 method. Inspection of the TS5 structure reveals that the bond breaking of O_3_–H_8_ and C_3_–H_4_ is stretched by 0.399 Å (41.26%) and 0.384 Å (34.88%), respectively. In comparison, the forming H_4_–H_8_ bond with a bond distance of 1.003 Å is longer than the hydrogen molecule (0.744 Å) in the TS5 structure, and the formation of the C_3_=O_3_ bond is shortened by 0.093 Å (6.55%). In other words, the O_3_–H_8_ and C_3_–H_4_ bond distances increase, showing the breaking of these bonds (0.967–1.366 Å) and (1.094–1.485 Å) in the related TSs, respectively. The C_3_–C_3_ bond distance reveals changes from single to double bond character (1.421–1.328 Å) in TSs. The H_4_–H_8_ bond is forming as the distance between these atoms decreases in the TS (1.003 Å).

As seen from Scheme 1 and Table 1, the removal of the CO_2_ molecule from the 3-HBA molecule occurs through the four-membered rings **TS6** with an imaginary frequency of 2032.1*i* at the CBS-QB3 method. In this chemical reaction, the hydrogen atom (H_1_) on the hydroxyl group on C_1_ is transferred to the C_2_ atom, and simultaneously, the C_1_–C_2_ bond is broken to produce CO_2_ (see Figure 5). The direct removal of the carboxyl group to produce carbon dioxide and isopropyl alcohol is the only exothermic reaction among all investigated channels [**R1**–**R6**]. Through the elimination of CO_2_, the formed double bond C_1_–O_2_ as well as the single bond C_2_–H_1_ is shortened by 0.094 Å (8.10%) and 0.19 Å (17.40%), respectively, while the broken C_1_–C_2_ and O_2_–H_1_ bonds stretch by 0.454 Å (30.09%) and 0.312 Å (32.17%), respectively.

The parameter *n*_T_ describes the position of the TS structure along the reaction coordinate^76^
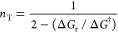
10According to this equation, when *n*_T_ < 0.5 (indicating an early TS), the TS structure is similar to the reactant; conversely, when *n*_T_ > 0.5, it resembles the product (suggesting a late TS).^33^ In the pyrolysis of 3-HBA through pathways **1**–**6**, the TS structures are identical to the 3-HBA and the associated products (**P1**–**P6**). By optimizing all stationary points along with reactions **1**–**6** and determining the activation and reaction Gibbs free energy, the *n*_T_ values for pathways **1**–**6** are approximately 0.52, 0.54, 0.44, 0.48, 0.44, and 0.74, respectively. This indicates that reactions **R3** and **R5**, with an *n*_T_ value of 0.44, closely resemble 3-HBA, while reaction **R6** resembles the related product (CO_2_ and CH_3_CHOHCH_3_).

Table 3 provides a summary of the change in standard thermodynamic parameters Δ*G*°, Δ*H*°, and Δ*S*° for the most favorable barrier reactions exhibiting lower energy barrier heights within the studied temperature ranges, as determined by the CBS-QB3 method. The results indicate that all investigated pathways exhibit positive entropy, and all thermodynamic parameters show an inverse relationship with Kelvin temperature; in other words, as the temperature increases, all thermodynamic values decrease. As can be seen from Table 3, pathway **R6** is identified as a spontaneous (Δ*G*° < 0) and exothermic (Δ*H*° < 0) reaction, while pathways **R3** and **R4** are characterized as spontaneous (Δ*G*° < 0) and endothermic (Δ*H*° > 0) reactions.

**Table 3:** Relative Thermodynamic Parameters (in kcal mol^–1^) as well as Reaction Entropies (in cal mol^–1^ K^–1^) for Main Barrier Reactions at Different Temperatures at the CBS-QB3 Method

	**R3**			**R4**			**R6**		
*T* (K)	Δ*H*°_298 K_	Δ*G*°_298 K_	Δ*S*°_298 K_	Δ*H*°_298 K_	Δ*G*°_298 K_	Δ*S*°_298 K_	Δ*H*°_298 K_	Δ*G*°_298 K_	Δ*S*°_298 K_
298	15.77	3.76	40.28	12.86	0.91	40.10	–6.48	–17.58	37.23
400	15.92	–0.37	40.74	13.03	–3.21	40.60	–6.54	–21.37	37.09
600	15.88	–8.52	40.67	12.97	–11.33	40.51	–6.90	–28.72	36.37
700	15.73	–12.58	40.44	12.81	–15.37	40.26	–7.15	–32.34	35.99
800	15.51	–16.61	40.16	12.58	–19.38	39.96	–7.42	–35.92	35.63
900	15.26	–20.61	39.85	12.31	–23.36	39.64	–7.69	–39.47	35.30
1000	14.96	–24.58	39.54	12.01	–27.31	39.32	–7.98	–42.98	35.00
1100	14.64	–28.52	39.24	11.68	–31.23	39.00	–8.26	–46.47	34.73
1200	14.30	–32.43	38.94	11.33	–35.11	38.70	–8.55	–49.93	34.48
1300	13.94	–36.31	38.65	10.97	–38.97	38.41	–8.83	–53.37	34.26
1400	13.57	–40.16	38.38	10.59	–42.79	38.13	–9.12	–56.78	34.04
1500	13.19	–43.98	38.12	10.21	–46.59	37.87	–9.40	–60.18	33.83
1600	12.80	–47.78	37.87	9.82	–50.37	37.62	–9.69	–63.55	33.66
1700	12.41	–51.56	37.63	9.42	–54.12	37.38	–9.97	–66.91	33.97

### 3.5 Thermochemistry of Simple Bond Fission Reactions

**Figure 8:**
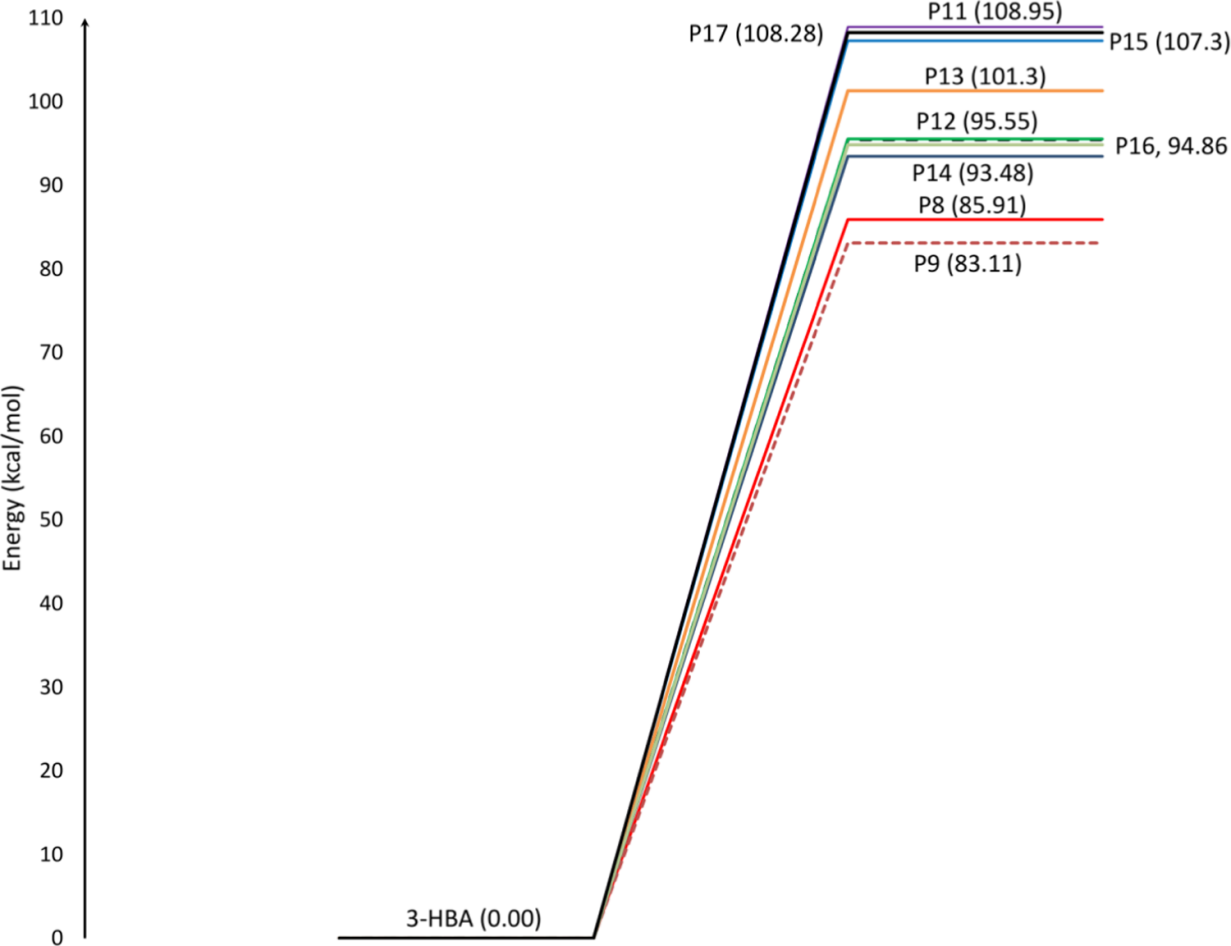
Potential energy profiles (Δ*E*_298 K_ and Δ*E*_298 K_^†^ are in kcal mol^–1^) for barrierless reactions involved in the unimolecular reactions of 3-HBA at the CBS-QB3 method (*T* = 298 K, *P* = 1 bar).

Table 4 presents thermodynamic data for various simple homolytic bond fission reactions (barrierless reactions), calculated using the M06-2X and CBS-QB3 methods. The potential energy profile is depicted in Figure 8. Inspection of different barrierless reactions during the decomposition of 3-HBA indicates that the production of ^•^CH_2_COOH and CH_3_C^•^HOH occurs through the cleavage of the C_2_–C_3_ bond, exhibiting the lowest endothermic energy (83.1 kcal mol^–1^) among all simple fission reactions.

**Table 4:** Energetic and Thermodynamics Parameters for the Considered Barrierless Reactions Encounter **3-HBA** Decomposition (in kcal mol^–1^) at the Studied Methods (*P* = 1 atm)

method	M06-2X/cc-pVTZ			CBS-QB3		
reaction	Δ*E*_0 K_	Δ*H*°_298 K_	Δ*G*°_298 K_	Δ*E*_0 K_	Δ*H*°_298 K_	Δ*G*°_298 K_
3-HBA	0.00	0.00	0.00	0.00	0.00	0.00
**R7**: 3-HBA → ^•^CH_3_ + ^•^CH(OH)CH_2_CO_2_H	84.47	86.29	72.65	85.91	87.60	74.33
**R8**: 3-HBA → CH_3_^•^CH(OH) + ^•^CH_2_CO_2_H	81.28	82.48	68.26	83.11	84.25	70.27
**R9**: 3-HBA → CH_3_CH(OH)^•^CH_2_ + ^•^CO_2_H	94.45	95.75	81.66	95.47	96.80	82.67
**R10**: 3-HBA → ^•^OH + CH_3_CH(OH)CH_2_^•^CO	109.64	111.37	99.25	108.95	110.65	98.74
**R11**: 3-HBA → ^•^OH + CH_3_CH^•^CH_2_CO_2_H	95.18	97.06	84.17	95.55	97.43	84.51
**R12**: 3-HBA → ^•^H + ^•^CH_2_CH(OH)CH_2_CO_2_H	99.20	100.83	92.02	101.30	102.96	94.09
**R13**: 3-HBA → ^•^H + CH_3_C^•^(OH)CH_2_CO_2_H	92.18	93.92	84.37	93.48	95.25	85.59
**R14**: 3-HBA → ^•^H + CH_3_CH(OH)^•^CHCO_2_H	105.14	106.61	97.67	107.30	108.82	98.52
**R15**: 3-HBA → ^•^H + CH_3_CH(O^•^)CH_2_CO_2_H	93.73	95.38	86.50	94.86	96.37	87.94
**R16**: 3-HBA → ^•^H + CH_3_CH(OH)CH_2_COO^•^	109.27	110.83	101.89	108.28	109.79	100.81

The production of hydroxyl radical through reaction **R11** can be accomplished via the thermal decomposition of the C_3_–O_3_ bond. The reaction requires a reaction energy of 95.18 (95.55) kcal mol^–1^ at the M06-2X (CBS-QB3) method, which is close to the obtained results for 2-butanol (95 kcal mol^–1^) and 2-methoxyethanol (97.3 kcal mol^–1^).^6,77^ The removal of the hydroxyl group via chemical reaction **R10** from the acidic COOH is the most costly endothermic reaction, with an energy of 109.64 (108.95) kcal mol^–1^ at the M06-2X (CBS-QB3) method. Due to the energy overlap between complex and simple bond cleavage reactions, simple homolytic bond fission reactions may compete with complex ones at higher temperatures.

### 3.6 Chemical Kinetic Simulations

Table 5 presents the rate constants for all barrier reactions [**R1**–**R6**] using the TST and RRKM theories incorporating the Eckert tunneling coefficient. These kinetic rate constants are calculated at a pressure of 1 bar over the temperature ranging from 600 to 1700 K. Table 6 provides the rate constants for simple homolytic bond fission reactions [**R7**–**R11**] under the same conditions.

**Table 5:** TST and RRKM (in Parentheses) Rate Constants (in s^–1^) for 3-HBA Pyrolysis Via Barrier Reactions over the Temperature Range 600–1700 K at the CBS-QB3 Method (*P* = 1 bar)

*T* (K)	*k*_**R1**_	*k*_**R2**_	*k*_**R3**_	*k*_**R4**_	*k*_**R5**_	*k*_**R6**_
600	3.52 × 10^–20^ (2.83 × 10^–20^)	5.87 × 10^–15^ (5.72 × 10^–15^)	1.53 × 10^–11^ (4.70 × 10^–12^)	1.32 × 10^–8^ (6.19 × 10^–9^)	4.42 × 10^–18^ (9.60 × 10^–19^)	3.50 × 10^–13^ (1.05 × 10^–13^)
650	1.31 × 10^–17^ (1.09 × 10^–17^)	9.51 × 10^–13^ (9.27 × 10^–13^)	1.14 × 10^–9^ (4.34 × 10^–10^)	6.16 × 10^–7^ (3.28 × 10^–7^)	9.09 × 10^–16^ (2.66 × 10^–16^)	3.43 × 10^–11^ (1.28 × 10^–11^)
700	2.09 × 10^–15^ (1.79 × 10^–15^)	7.42 × 10^–11^ (7.30 × 10^–11^)	4.77 × 10^–8^ (2.21 × 10^–8^)	1.69 × 10^–5^ (9.94 × 10^–6^)	9.16 × 10^–14^ (3.32 × 10^–14^)	1.81 × 10^–9^ (7.93 × 10^–10^)
750	1.72 × 10^–13^ (1.50 × 10^–13^)	3.28 × 10^–9^ (3.22 × 10^–9^)	1.23 × 10^–6^ (6.18 × 10^–7^)	3.04 × 10^–4^ (1.92 × 10^–4^)	5.16 × 10^–12^ (2.19 × 10^–12^)	5.73 × 10^–8^ (2.84 × 10^–8^)
800	8.20 × 10^–12^ (7.27 × 10^–12^)	9.06 × 10^–8^ (8.90 × 10^–8^)	2.15 × 10^–5^ (1.19 × 10^–5^)	3.84 × 10^–3^ (2.57 × 10^–3^)	1.80 × 10^–10^ (8.59 × 10^–11^)	1.20 × 10^–6^ (6.53 × 10^–7^)
850	2.48 × 10^–10^ (2.24 × 10^–10^)	1.68 × 10^–6^ (1.67 × 10^–6^)	2.75 × 10^–4^ (1.63 × 10^–4^)	3.60 × 10^–2^ (2.54 × 10^–2^)	4.17 × 10^–9^ (2.19 × 10^–9^)	1.76 × 10^–5^ (1.04 × 10^–5^)
900	5.19 × 10^–9^ (4.73 × 10^–9^)	2.28 × 10^–5^ (2.26 × 10^–5^)	2.64 × 10^–3^ (1.67 × 10^–3^)	2.68 × 10^–1^ (1.96 × 10^–1^)	6.92 × 10^–8^ (3.93 × 10^–8^)	1.95 × 10^–4^ (1.23 × 10^–4^)
950	7.90 × 10^–8^ (7.25 × 10^–8^)	2.37 × 10^–4^ (2.34 × 10^–4^)	2.02 × 10^–2^ (1.34 × 10^–2^)	1.61 × 10° (1.22 × 10°)	8.60 × 10^–7^ (5.21 × 10^–7^)	1.70 × 10^–3^ (1.12 × 10^–3^)
1000	9.17 × 10^–7^ (8.49 × 10^–7^)	1.94 × 10^–3^ (1.92 × 10^–3^)	1.27 × 10^–1^ (8.77 × 10^–2^)	8.11 × 10° (6.33 × 10°)	8.36 × 10^–6^ (5.34 × 10^–6^)	1.18 × 10^–2^ (8.18 × 10^–3^)
1050	8.43 × 10^–6^ (7.88 × 10^–6^)	1.29 × 10^–2^ (1.29 × 10^–2^)	6.73 × 10^–1^ (4.81 × 10^–1^)	3.53 × 10^1^ (2.82 × 10^1^)	6.58 × 10^–5^ (4.40 × 10^–5^)	6.92 × 10^–2^ (4.96 × 10^–2^)
1100	6.35 × 10^–5^ (5.98 × 10^–5^)	7.30 × 10^–2^ (7.29 × 10^–2^)	3.05 × 10° (2.27 × 10°)	1.35 × 10^2^ (1.10 × 10^2^)	4.32 × 10^–4^ (3.00 × 10^–4^)	3.44 × 10^–1^ (2.56 × 10^–1^)
1150	4.01 × 10^–4^ (3.81 × 10^–4^)	3.56 × 10^–1^ (3.55 × 10^–1^)	1.23 × 10^1^ (9.34 × 10°)	4.56 × 10^2^ (3.79 × 10^2^)	2.41 × 10^–3^ (1.74 × 10^–3^)	1.50 × 10° (1.14 × 10°)
1200	2.19 × 10^–3^ (2.08 × 10^–3^)	1.53 × 10° (1.52 × 10°)	4.39 × 10^1^ (3.43 × 10^1^)	1.40 × 10^3^ (1.19 × 10^3^)	1.17 × 10^–2^ (8.69 × 10^–3^)	5.84 × 10° (4.53 × 10°)
1250	1.04 × 10^–2^ (9.96 × 10^–3^)	5.79 × 10° (5.78 × 10°)	1.44 × 10^2^ (1.13 × 10^2^)	3.98 × 10^3^ (3.38 × 10^3^)	5.04 × 10^–2^ (3.82 × 10^–2^)	2.02 × 10^1^ (1.61 × 10^1^)
1300	4.40 × 10^–2^ (4.23 × 10^–2^)	1.99 × 10^1^ (1.99 × 10^1^)	4.25 × 10^2^ (3.43 × 10^2^)	1.03 × 10^4^ (8.91 × 10^3^)	1.94 × 10^–1^ (1.51 × 10^–1^)	6.43 × 10^1^ (5.18 × 10^1^)
1350	1.68 × 10^–1^ (1.61 × 10^–1^)	6.25 × 10^1^ (6.23 × 10^1^)	1.17 × 10^3^ (9.54 × 10^2^)	2.52 × 10^4^ (2.18 × 10^4^)	6.81 × 10^–1^ (5.36 × 10^–1^)	1.87 × 10^2^ (1.53 × 10^2^)
1400	5.77 × 10^–1^ (5.59 × 10^–1^)	1.81 × 10^2^ (1.80 × 10^2^)	2.98 × 10^3^ (2.47 × 10^3^)	5.75 × 10^4^ (5.01 × 10^4^)	2.16 × 10° (1.74 × 10°)	5.05 × 10^2^ (4.20 × 10^2^)
1450	1.84 × 10° (1.78 × 10°)	4.88 × 10^2^ (4.85 × 10^2^)	7.10 × 10^3^ (5.99 × 10^3^)	1.24 × 10^5^ (1.09 × 10^5^)	6.40 × 10° (5.24 × 10°)	1.27 × 10^3^ (1.07 × 10^3^)
1500	5.43 × 10° (5.26 × 10°)	1.23 × 10^3^ (1.22 × 10^3^)	1.62 × 10^4^ (1.37 × 10^4^)	2.54 × 10^5^ (2.23 × 10^5^)	1.77 × 10^1^ (1.46 × 10^1^)	3.03 × 10^3^ (2.58 × 10^3^)
1550	1.48 × 10^1^ (1.45 × 10^1^)	2.92 × 10^3^ (2.90 × 10^3^)	3.47 × 10^4^ (2.97 × 10^4^)	4.98 × 10^5^ (4.37 × 10^5^)	4.57 × 10^1^ (3.83 × 10^1^)	6.83 × 10^3^ (5.85 × 10^3^)
1600	3.83 × 10^1^ (3.74 × 10^1^)	6.58 × 10^3^ (6.52 × 10^3^)	7.15 × 10^4^ (6.18 × 10^4^)	9.45 × 10^5^ (8.20 × 10^5^)	1.12 × 10^2^ (9.44 × 10^2^)	1.46 × 10^4^ (1.26 × 10^4^)
1650	9.36 × 10^1^ (9.14 × 10^1^)	1.41 × 10^4^ (1.39 × 10^4^)	1.40 × 10^5^ (1.21 × 10^5^)	1.71 × 10^6^ (1.47 × 10^6^)	2.58 × 10^2^ (2.21 × 10^2^)	2.99 × 10^4^ (2.60 × 10^4^)
1700	2.17 × 10^2^ (2.12 × 10^2^)	2.89 × 10^4^ (2.85 × 10^4^)	2.66 × 10^5^ (2.29 × 10^5^)	3.00 × 10^6^ (2.55 × 10^6^)	5.71 × 10^2^ (4.90 × 10^2^)	5.87 × 10^4^ (5.13 × 10^4^)

**Table 6:** Calculated Rate Constants for Simple Homolytic Bond Fission Reactions [**R7**–**R11**] Based on the Computed CBS-QB3 Energies (*P* = 1 bar)

*T* (K)	*k*_**R7**_	*k*_**R8**_	*k*_**R9**_	*k*_**R10**_	*k*_**R11**_
600	3.09 × 10^–11^	1.23 × 10^–9^	2.95 × 10^–13^	2.11 × 10^–20^	6.02 × 10^–15^
650	8.06 × 10^–9^	2.64 × 10^–7^	1.42 × 10^–10^	2.54 × 10^–17^	3.10 × 10^–12^
700	9.41 × 10^–7^	2.60 × 10^–5^	2.80 × 10^–8^	1.10 × 10^–14^	6.50 × 10^–10^
750	5.77 × 10^–5^	1.39 × 10^–3^	2.72 × 10^–6^	2.12 × 10^–12^	6.66 × 10^–8^
800	2.10 × 10^–3^	4.45 × 10^–2^	1.48 × 10^–4^	2.10 × 10^–10^	3.78 × 10^–6^
850	4.96 × 10^–2^	9.45 × 10^–1^	4.98 × 10^–3^	1.21 × 10^–8^	1.33 × 10^–4^
900	8.20 × 10^–1^	1.42 × 10^1^	1.13 × 10^–1^	4.39 × 10^–7^	3.15 × 10^–3^
950	1.00 × 10^1^	1.59 × 10^2^	1.82 × 10°	1.09 × 10^–5^	5.32 × 10^–2^
1000	9.46 × 10^1^	1.39 × 10^3^	2.22 × 10^1^	1.96 × 10^–4^	6.72 × 10^–1^
1050	7.16 × 10^2^	9.86 × 10^3^	2.13 × 10^2^	2.66 × 10^–3^	6.66 × 10°
1100	4.48 × 10^3^	5.82 × 10^4^	1.64 × 10^3^	2.83 × 10^–2^	5.31 × 10^1^
1150	2.38 × 10^4^	2.92 × 10^5^	1.06 × 10^4^	2.44 × 10^–1^	3.55 × 10^2^
1200	1.09 × 10^5^	1.28 × 10^6^	5.83 × 10^4^	1.76 × 10°	2.00 × 10^3^
1250	4.43 × 10^5^	4.97 × 10^6^	2.79 × 10^5^	1.08 × 10^1^	9.84 × 10^3^
1300	1.60 × 10^6^	1.73 × 10^7^	2.11 × 10^6^	5.71 × 10^1^	4.27 × 10^4^
1350	5.25 × 10^6^	5.47 × 10^7^	4.44 × 10^6^	2.68 × 10^2^	1.66 × 10^5^
1400	1.57 × 10^7^	1.58 × 10^8^	1.52 × 10^7^	1.12 × 10^3^	5.81 × 10^5^
1450	4.35 × 10^7^	4.26 × 10^8^	4.78 × 10^7^	4.24 × 10^3^	1.87 × 10^6^
1500	1.13 × 10^8^	1.07 × 10^9^	1.38 × 10^8^	2.19 × 10^4^	5.52 × 10^6^
1550	2.71 × 10^8^	2.51 × 10^9^	3.74 × 10^8^	4.63 × 10^4^	1.52 × 10^7^
1600	6.18 × 10^8^	5.61 × 10^9^	9.45 × 10^8^	1.37 × 10^5^	3.93 × 10^7^
1650	1.34 × 10^9^	1.19 × 10^10^	2.25 × 10^9^	3.77 × 10^5^	1.49 × 10^8^
1700	2.75 × 10^9^	2.39 × 10^10^	5.09 × 10^9^	9.75 × 10^5^	2.21 × 10^8^

The obtained results in Table 5 demonstrate a good agreement between the TST and RRKM rate constants, with individual rate constants increasing as the temperature rises, indicating a positive temperature dependency. The accompanied Eckert tunneling corrections during TST calculations are given in Table S4 in the SI. These results suggest that tunneling is effective for pathways **R3**–**R6** up to 1400 K, while it is considered negligible for other reactions within the applied temperature range.

The branching ratio of the main routes of 3-HBA pyrolysis within a temperature range of 600–1700 K is detailed in Table 7. The outcomes reveal the predominant occurrence of water elimination through reaction pathway **R4** [3-HBA → H_2_O+CH_3_CH=CHCO_2_H], leading to the formation of 2-butenoic acid, with a minor contribution from reaction pathway **R8** [3-HBA → CH_3_^•^CH(OH)+^•^CH_2_CO_2_H] at *T* ≤ 650 K. Above 700 K, reaction **R8** becomes the primary decomposition route, with a small contribution from reaction pathway **R9** [3-HBA → CH_3_CH(OH)^•^CH_2_+^•^CO_2_H] and reaction **R7** [3-HBA → ^•^CH_3_+^•^CH(OH)CH_2_CO_2_H**]** which are approximately 16 and 9%, respectively, at 1700 K.

**Table 7:** Branching Ratio (Γ) of the Main Pathways **R3**, **R4**, **R7**, **R8**, **R9**, and **R11** in the Pyrolysis Process of 3-Hydroxybutyric Acid

*T* (K)	**R3**	**R4**	**R7**	**R8**	**R9**	**R11**
600	0.11	91.15	0.21	8.53	0.00	0.00
650	0.13	69.22	0.91	29.72	0.02	0.00
700	0.11	38.46	2.14	59.22	0.06	0.00
750	0.07	17.34	3.29	79.13	0.15	0.00
800	0.04	7.58	4.16	87.91	0.29	0.01
850	0.03	3.48	4.79	91.21	0.48	0.01
900	0.02	1.74	5.33	92.15	0.73	0.02
950	0.01	0.93	5.81	92.16	1.06	0.03
1000	0.01	0.53	6.23	91.72	1.46	0.04
1050	0.01	0.33	6.61	91.03	1.96	0.06
1100	0.00	0.21	6.94	90.22	2.54	0.08
1150	0.00	0.14	7.27	89.24	3.24	0.11
1200	0.00	0.10	7.53	88.21	4.02	0.14
1250	0.00	0.07	7.77	87.09	4.89	0.17
1300	0.00	0.05	7.59	82.13	10.03	0.20
1350	0.00	0.04	8.13	84.69	6.89	0.26
1400	0.00	0.03	8.29	83.33	8.04	0.31
1450	0.00	0.02	8.39	82.03	9.20	0.36
1500	0.00	0.02	8.50	80.62	10.44	0.42
1550	0.00	0.02	8.54	79.18	11.78	0.48
1600	0.00	0.01	8.57	77.76	13.11	0.54
1650	0.00	0.01	8.54	76.08	14.41	0.95
1700	0.00	0.01	8.61	74.78	15.91	0.69

## 4 Conclusions

The pyrolysis of 3-hydroxybutyric acid (3-HBA) in the gas phase was computationally investigated using density functional theory (M06-2X) in conjunction with the correlation consistent polarized valence triplet ζ (cc-pVTZ) basis set, as well as the CBS-QB3 composite method. Energy profiles were obtained and supplemented with calculations of rate coefficients and branching ratios at a pressure of 1 bar using conventional transition state theory (TST) and statistical Rice–Ramsperger–Kassel–Marcus (RRKM) theory. This study specifically focused on the analysis of simple and complex bond fission unimolecular reactions at a pressure of 1 bar across a temperature range from 600 to 1700 K. The obtained results can be summarized as follows:

[1]. At low temperatures, the most kinetically favorable reaction in 3-HBA pyrolysis is the removal of a water molecule through 1,3-hydrogen transfer to produce 2-butenoic acid. Meanwhile, the carbon dioxide elimination pathway is more thermodynamically favorable compared to other reactions.
[2]. At higher temperatures, the main route shifts to the simple homolytic bond fission of C_2_–C_3_ through reaction **R7** [i.e., 3-HBA → ^•^CH_3_ + ^•^CH(OH)CH_2_CO_2_H].
[3]. All available chemical reactions involved in the pyrolysis of 3-HBA are endothermic, except for the release of CO_2_ molecules [i.e., 3-HBA → CO_2_ + CH_3_CHOHCH_3_].
[4]. The elimination of the hydroxyl radical of the alcohol is 13.3 kcal mol^–1^ easier than that of the carboxylic acid.

## Supporting Information Available

The optimized geometry of 3-HBA and transition state structures using the CBS-QB3 method (Table S1); the standard energies and relative energies of all examined conformers of 3-HBA at the M06-2X/cc-pVTZ and CBS-QB3 methods (Table S2); bond lengths (Å) and bond angles (°) for 3-HBA and its TSs using CBS-QB3 and M06-2X/cc-pVTZ (in parentheses) theoretical methods (Table S3); Eckart tunneling corrections for the studied chemical reactions over the temperature range 600–1700 K at the CBS-QB3 method (Table S4); change of bond lengths along reaction coordinates for the formation of different products at the M06-2X/cc-pVTZ level of theory (Figure S1) (PDF)
