# Supplementary material for: Screening Stability, Thermochemistry, and Chemical Kinetics of 3-Hydroxybutanoic Acid as a Bifunctional Biodiesel Additive
Source: J Phys Chem A. 2024 May 10;128(20):4068–82. doi: 10.1021/acs.jpca.4c01338 (PMC11129312; doi:10.1021/acs.jpca.4c01338)
Supplement: Supplementary file 1 — jp4c01338_si_001.pdf [file jp4c01338_si_001.pdf]

Supporting Information

Screening Stability, Thermochemistry, and Chemical Kinetics of 3-Hydroxybutanoic Acid as a Bi-functional Biodiesel Additives

Mohamed A. Abdel-Rahman[1*], Abolfazl Shiroudi[2,3*], Jacek Czub[2,3], and Hao Zhao[4]

[1] Chemistry Department, Faculty of Science, Suez University, Suez 43518, Egypt

[2] Department of Physical Chemistry, Gdańsk University of Technology, Narutowicza 11/12, Gdańsk 80-233, Poland

[3] BioTechMed Center, Gdańsk University of Technology, Gdańsk 80-233, Poland

[4] College of Engineering, Peking University, Beijing, 100871, China

Contents

**Table S1**.	The optimized geometry of 3-HBA and transition state structures using the CBS-QB3 method.	S2
**Table S2.**	The standard energies and relative energies of all examined conformers of 3-HBA at the M06-2X/cc-pVTZ and CBS-QB3 methods.	S5
**Table S3.**	Bond lengths (Å) and bond angles (°) for 3-HBA and its TSs using CBS-QB3 and M06-2X/cc-pVTZ (in parentheses) theoretical methods.	S6
**Table S4.**	Eckart tunneling corrections for the studied chemical reactions over the temperature range 600–1700 K at the CBS-QB3 method.	S6
**Figure S1.**	Change of bond lengths along reaction coordinates for the formation of different products at the M06-2X/cc-pVTZ level of theory.	S8

* Corresponding authors:
E-mails: Mohamed.Abdel-Rahman@sci.suezuni.edu.eg; mohammadadel2015@yahoo.com (M.A. Abdel-Rahman); abolfazl.shiroudi@pg.edu.pl (A. Shiroudi)

Table S1. The optimized geometry of 3-HBA and transition state structures using the CBS-QB3 method.

3-HBA

C	-1.33755300	-0.01899300	-0.01917400
O	-2.41247400	-0.82603000	0.08772300
O	-1.44354600	1.16542800	-0.24666700
H	-3.19566500	-0.26684600	-0.03017600
C	-0.04023700	-0.76140600	0.19019500
H	0.06087500	-0.94063800	1.26725400
H	-0.11715300	-1.74105700	-0.28954700
C	1.18252900	0.02414400	-0.31142400
O	1.30818200	1.26189300	0.37448900
H	1.05277700	0.20506900	-1.38943500
C	2.47535200	-0.74707900	-0.09084400
H	3.31984400	-0.15947500	-0.45525000
H	2.46229500	-1.70498600	-0.61750400
H	2.62708500	-0.93285400	0.97593500
H	0.49210500	1.75045200	0.20184600

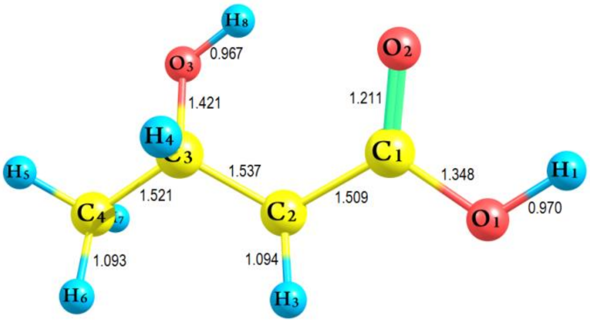

CBS-QB3 (0 K)= -382.362388
CBS-QB3 Energy= -382.354623
CBS-QB3 Enthalpy= -382.353678
CBS-QB3 Free Energy= -382.394732

TS1

C	1.35839300	0.11417900	0.09400200
O	2.38358200	-0.21185600	-0.43897700
O	0.90493500	1.39071900	0.09901300
H	1.53164200	1.91252600	-0.42473700
C	0.43327500	-0.81442200	0.85472800
H	0.92726900	-1.79306700	0.88166600
H	0.33704900	-0.46967700	1.88868200
C	-0.93300500	-0.97703700	0.24203600
O	-1.11356300	-0.75275800	-1.02793000
H	-1.62494400	-1.63369500	0.76863700
C	-2.43069000	0.94938600	0.16188200
H	-1.37938200	1.15800300	0.09540800
H	-3.02938700	1.73715000	0.61334900
H	-2.91395700	-0.01053600	0.13536800
H	-1.81576500	0.05782400	-1.13111000

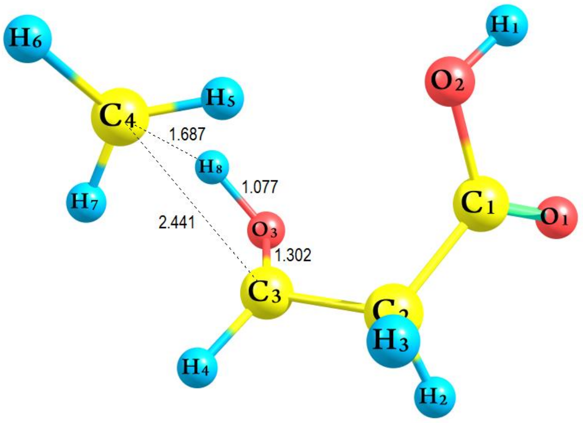

CBS-QB3 (0 K)= -382.218093
CBS-QB3 Energy= -382.210020
CBS-QB3 Enthalpy= -382.209076
CBS-QB3 Free Energy= -382.250916

TS2

C	-1.29018900	-0.18223200	0.18623200
O	-0.88213500	-1.31321800	0.50329500
O	-1.95261800	-0.04531000	-1.01859100
H	-1.99077200	-0.94324200	-1.37419300
C	-1.07648900	1.04336700	0.86920300
H	-1.66758300	1.89628700	0.55054600
H	-0.91371000	0.95628400	1.93844300
C	1.54344500	0.29531400	0.27203300
O	1.41710900	1.21735400	-0.61516900
H	1.14608600	0.49059100	1.26059100
C	2.09456600	-0.99593700	-0.09183100
H	1.15890300	-1.60228800	-0.14170300
H	2.69939900	-1.43527700	0.70245700
H	2.59327600	-0.99276300	-1.05990900
H	0.68756000	1.79672500	-0.24634300

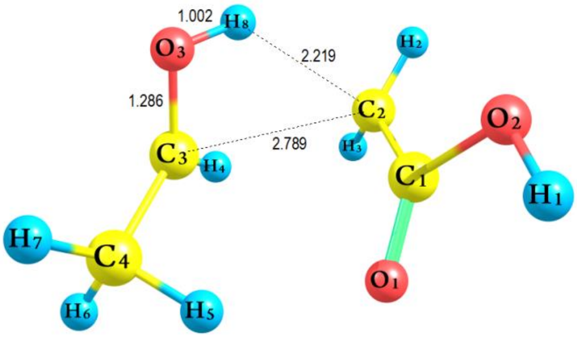

CBS-QB3 (0 K)= -382.239624
CBS-QB3 Energy= -382.231296
CBS-QB3 Enthalpy= -382.230351
CBS-QB3 Free Energy= -382.273146

TS3

C	-1.24667600	-0.07530400	0.09990600
O	-2.03111300	-0.85925000	-0.35969600
O	-1.25845900	1.24683500	-0.22856800
H	-1.96937800	1.36911100	-0.87627200
C	-0.14530600	-0.39064600	1.08988600
H	-0.15245400	0.35553900	1.88653600
H	-0.37814800	-1.36814000	1.52112100
C	1.23744900	-0.49127800	0.47612500
O	1.65679300	1.24727700	-0.08739400
H	2.02827800	-0.52087100	1.21707800
C	1.47919100	-1.04865900	-0.81195800
H	1.69088000	0.35277100	-0.96833500
H	0.62958200	-1.43280500	-1.36798100
H	2.41056000	-1.58574200	-0.95462300
H	0.85496600	1.78656400	-0.17602000

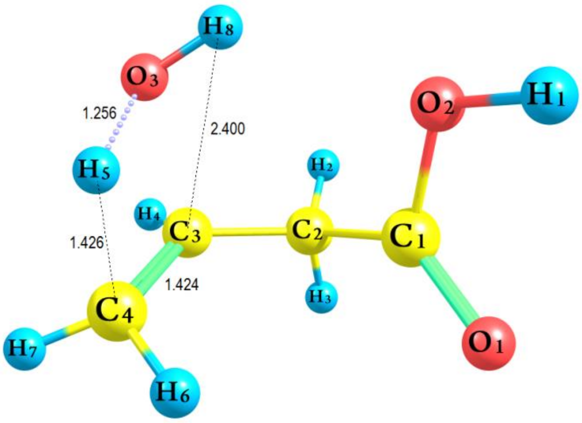

CBS-QB3 (0 K)= -382.253068
CBS-QB3 Energy= -382.245234
CBS-QB3 Enthalpy= -382.244290
CBS-QB3 Free Energy= -382.285464

TS4

C	-1.32236000	-0.07008100	-0.07557100
O	-2.48249000	-0.30717300	-0.33003800
O	-1.00827000	0.93782500	0.82744600
H	-1.86269300	1.31015700	1.08723300
C	-0.16101400	-0.80136200	-0.54684900
H	0.57637100	-1.30400400	0.68002100
H	-0.39757900	-1.56738600	-1.27464300
C	1.20434000	-0.27026200	-0.57160600
O	1.66173600	-0.91660600	0.94804500
H	1.85992200	-0.86974800	-1.19702600
C	1.59146000	1.18754400	-0.57838100
H	1.01429200	1.76671800	0.13944100
H	1.37120500	1.58043700	-1.57610900
H	2.66093400	1.31187000	-0.39614400
H	1.53518800	-0.21544800	1.60804900

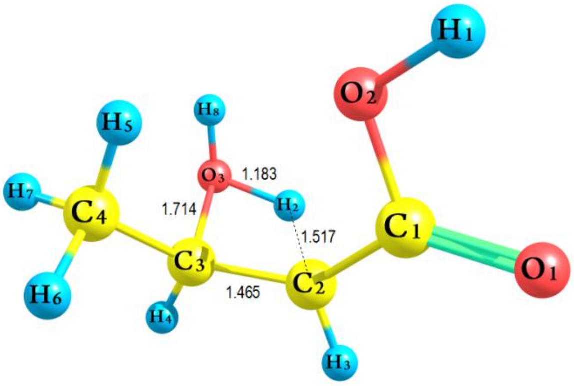

CBS-QB3 (0 K)= -382.266676
CBS-QB3 Energy= -382.258753
CBS-QB3 Enthalpy= -382.257809
CBS-QB3 Free Energy -382.299459

TS5

C	1.22707100	0.19953700	0.07905800
O	1.23297500	1.30469900	-0.39962500
O	2.25179700	-0.66542900	-0.05440400
H	2.92211700	-0.22139100	-0.59671900
C	0.10477600	-0.38720600	0.92072500
H	0.32686600	-1.42959000	1.13766400
H	0.04833300	0.18133500	1.85324300
C	-1.21576200	-0.30594900	0.13410200
O	-1.31934200	-1.10925900	-0.91886300
H	-2.08731600	-0.94946300	1.14959400
C	-1.86954300	1.05631500	0.05858100
H	-1.22713500	1.66075100	-0.58779600
H	-1.94479600	1.54427200	1.03256200
H	-2.85185100	0.96578000	-0.40324000
H	-1.98890900	-1.36795500	0.24303100

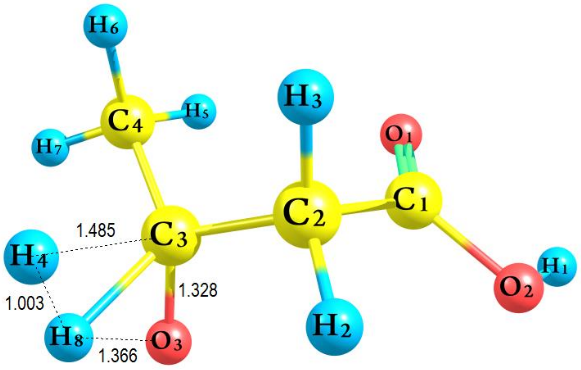

CBS-QB3 (0 K)= -382.225354
CBS-QB3 Energy= -382.217591
CBS-QB3 Enthalpy= -382.216647
CBS-QB3 Free Energy= -382.258150

TS6

C	1.42989300	0.26933400	-0.05606900
O	1.33938900	1.39795800	-0.36612500
O	2.14088600	-0.75936500	-0.15388900
H	1.08082500	-1.02861700	0.51432700
C	-0.02069300	-0.39530000	1.08659000
H	0.06042000	-1.37256300	1.60561600
H	0.06682300	0.35624500	1.86953600
C	-1.33111200	-0.35059300	0.30874500
O	-1.08451100	-1.10423200	-0.88568700
H	-2.10470400	-0.86182400	0.90159900
C	-1.82680200	1.06108900	-0.00438000
H	-1.10218000	1.60759400	-0.60721400
H	-2.01136500	1.62232400	0.91629400
H	-2.77250700	1.01037600	-0.55342600
H	-1.89113700	-1.11560900	-1.41044400

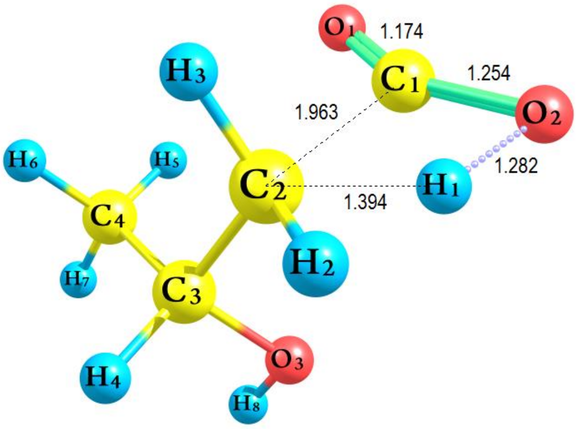

CBS-QB3 (0 K)=	-382.246360
CBS-QB3 Energy=	-382.238111
CBS-QB3 Enthalpy=	-382.237167
CBS-QB3 Free Energy=	-382.279354

Table S2. The standard energies and relative energies of all examined conformers of 3-HBA at the M06-2X/cc-pVTZ and CBS-QB3 methods.

Conformer \ method	CBS-QB3		M06-2X/cc-pVTZ	
	CBS-QB3 (0 K) (a.u.)	ΔE (kcal/mol)	E_{elec}+ZPVE (a.u.)	ΔE (kcal/mol)
A	-382.366680	0.000	-382.805956	0.000
B	-382.363098	2.248	-382.801775	2.624
C	-382.364954	1.083	-382.804449	0.946
D	-382.362945	2.344	-382.801922	2.531
E	-382.361519	3.239	-382.799855	3.828
F	-382.362457	2.650	-382.801570	2.752

Table S3. Bond lengths (Å) and bond angles (°) for 3-HBA and its TSs using CBS-QB3 and M06-2X/cc-pVTZ (in parentheses) theoretical methods.

Parameters	**3-HBA**	**TS1**	**TS2**	**TS3**	**TS4**	**TS5**	**TS6**
$C_1–C_2$	1.510 (1.502)	1.515 (1.506)	1.419 (1.465)	1.514 (1.505)	1.451 (1.433)	1.520 (1.513)	1.962 (1.918)
$C_2–C_3$	1.547 (1.538)	1.506 (1.507)	2.789 (2.135)	1.516 (1.513)	1.465 (1.474)	1.539 (1.530)	1.524 (1.519)
$C_3–C_4$	1.530 (1.521)	2.441 (2.316)	1.450 (1.485)	1.424 (1.423)	1.508 (1.509)	1.513 (1.505)	1.529 (1.519)
$C_1–O_2$	1.369 (1.358)	1.355 (1.346)	1.382 (1.353)	1.362 (1.355)	1.389 (1.397)	1.348 (1.337)	1.254 (1.247)
$C_3–O_3$	1.422 (1.411)	1.302 (1.287)	1.286 (1.303)	1.875 (1.783)	1.713 (1.603)	1.328 (1.321)	1.434 (1.422)
$C_1–C_2–C_3$	115.24 (112.97)	114.56 (112.54)	78.86 (106.93)	114.36 (112.85)	124.65 (121.22)	109.27 (107.02)	109.17 (107.08)
$C_2–C_3–C_4$	113.11 (112.13)	118.99 (116.03)	130.50 (104.20)	123.16 (121.62)	126.13 (124.73)	116.35 (116.17)	114.23 (113.80)
$O_2–C_1–C_2$	111.95 (111.97)	111.79 (111.81)	113.97 (112.14)	111.47 (111.71)	113.32 (112.25)	111.62 (112.26)	100.76 (102.27)
$O_3–C_3–C_2$	111.84 (111.86)	120.26 (120.28)	82.13 (97.21)	105.31 (105.45)	95.57 (97.90)	116.11 (115.91)	105.18 (105.16)
$O_3–C_3–C_4$	111.76 (111.70)	75.35 (77.15)	120.18 (120.91)	93.04 (95.76)	107.46 (108.07)	118.11 (118.16)	111.75 (111.82)

Table S4. Eckart tunneling corrections for the studied chemical reactions over the temperature range 600–1700 K at the CBS-QB3 method.

T (K)	**R1**	**R2**	**R3**	**R4**	**R5**	**R6**
600	1.26	1.04	3.31	2.16	4.68	3.4
650	1.22	1.04	2.67	1.9	3.47	2.72
700	1.18	1.03	2.28	1.72	2.8	2.31
750	1.16	1.03	2.02	1.6	2.39	2.04
800	1.14	1.02	1.84	1.51	2.12	1.85
850	1.12	1.02	1.71	1.43	1.92	1.71
900	1.11	1.02	1.6	1.38	1.78	1.61
950	1.1	1.02	1.52	1.33	1.67	1.53
1000	1.09	1.02	1.46	1.29	1.58	1.46
1050	1.08	1.01	1.41	1.26	1.51	1.41
1100	1.07	1.01	1.36	1.24	1.45	1.36
1150	1.06	1.01	1.33	1.21	1.4	1.33
1200	1.06	1.01	1.29	1.19	1.36	1.3
1250	1.05	1.01	1.27	1.18	1.33	1.27
1300	1.05	1.01	1.25	1.16	1.3	1.25
1350	1.05	1.01	1.23	1.15	1.28	1.23
1400	1.04	1.01	1.21	1.14	1.25	1.21
1450	1.04	1.01	1.19	1.13	1.23	1.19
1500	1.04	1.01	1.18	1.12	1.22	1.18
1550	1.03	1.01	1.17	1.11	1.2	1.17
1600	1.03	1.01	1.16	1.11	1.19	1.16
1650	1.03	1.01	1.15	1.1	1.18	1.15
1700	1.03	1.01	1.14	1.1	1.17	1.14

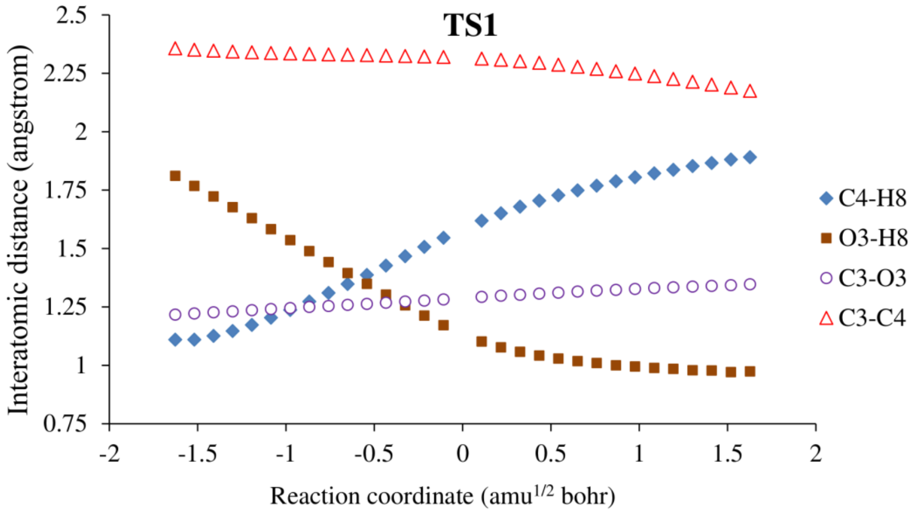

Fig. S1a. Formation of methane and 3-oxopropionic acid (via **TS1**).

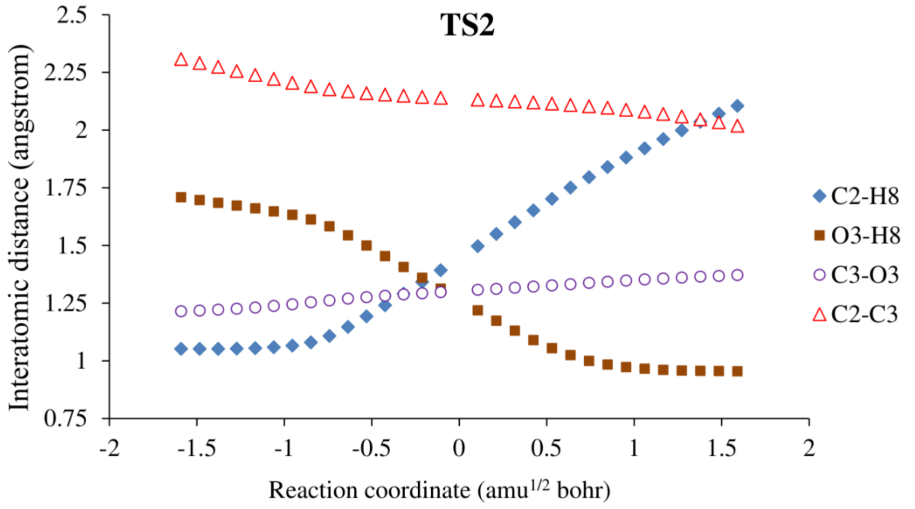

Fig. S1b. Formation of acetic acid and acetaldehyde (via **TS2**).

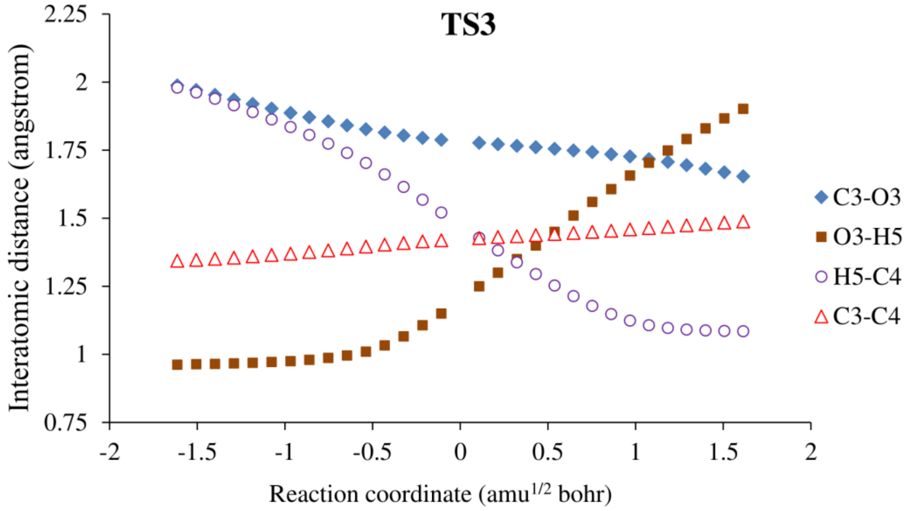

Fig. S1c. Formation of H_2O and 3-butenoic acid (via **TS3**).

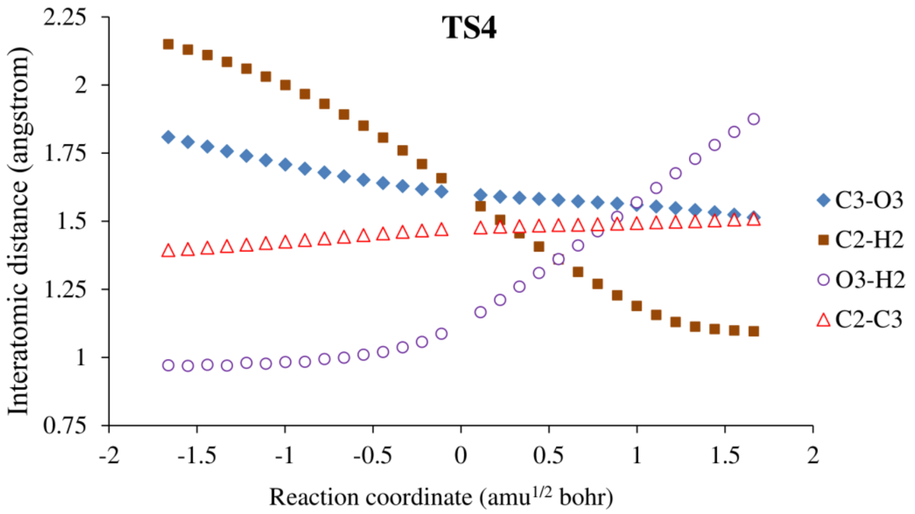

Fig. S1d. Formation of H_2O and 2-butenoic acid (via **TS4**).

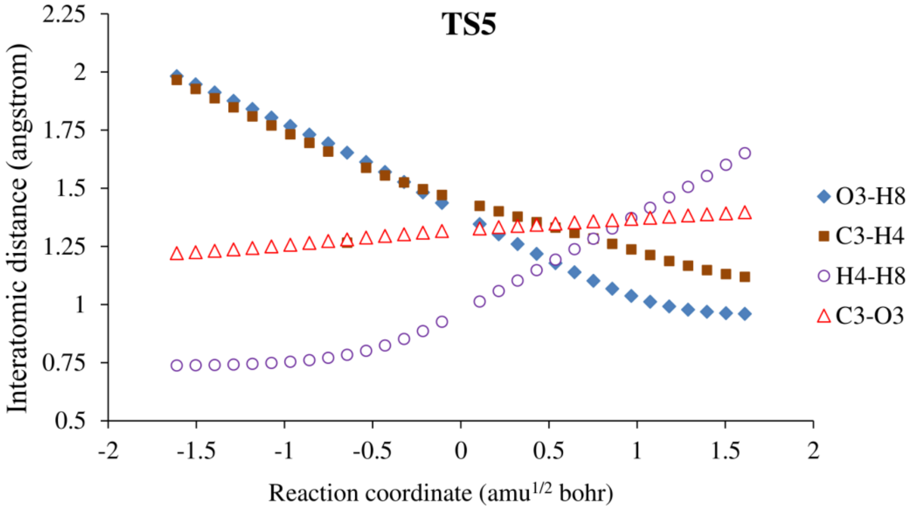

Fig. S1e. Formation of acetoacetic acid and H_2 (via **TS5**).

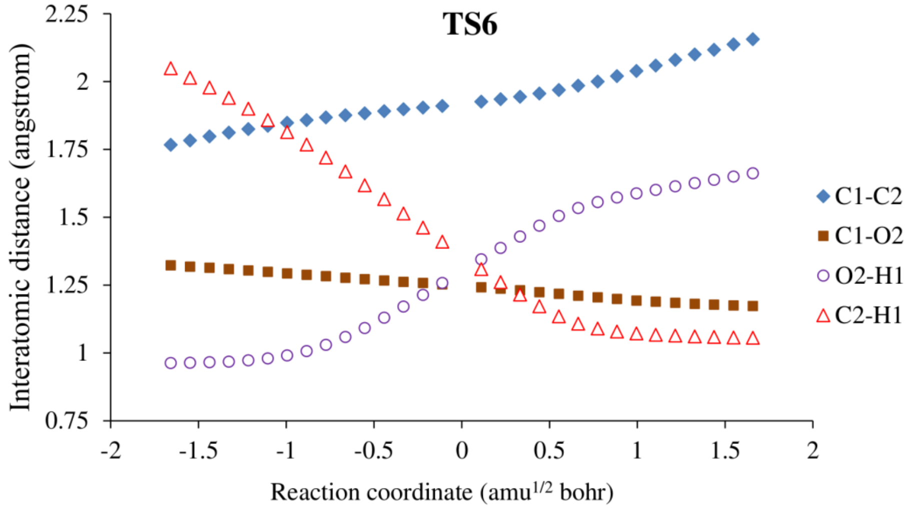

Fig. S1f. Formation of CO_2 and isopropyl alcohol (via **TS6**).

Figure S1. Change of bond lengths along reaction coordinates for the formation of different products at the M06-2X/cc-pVTZ level of theory.
